# Supplementary material for: Pulcherriminic acid biosynthesis and transport: insights from a heterologous system in Saccharomyces cerevisiae
Source: FEMS Yeast Res. 2025 Jul 28;25:foaf039. doi: 10.1093/femsyr/foaf039 (PMC12342472; doi:10.1093/femsyr/foaf039)
Supplement: foaf039_Supplemental_File [file foaf039_supplemental_file.docx]

**Supplementary Material**

**Pulcherriminic acid Biosynthesis and Transport: Insights from a heterologous system in *Saccharomyces cerevisiae***

Alicia Maciá Valero^1^, Jeroen J. van Wageningen^1^, Alexander J. Foster^1^, Ana Rita Oliveira^2^, Clemens Mayer^2^ and Sonja Billerbeck^1,3*^

^1^*Molecular Microbiology, Groningen Biomolecular Sciences and Biotechnology Institute, University of Groningen, 9747 AG Groningen, the Netherlands.*

*^2^Biomolecular Chemistry and Catalysis, Stratingh Institute for Chemistry, University of Groningen, 9747 AG Groningen, the Netherlands.*

*^3^Department of Bioengineering, Imperial College London, South Kensington Campus, SW7 2AZ, London, UK*

^*^Corresponding author: s.billerbeck@imperial.ac.uk

**Supplementary Table 1**. Strains used in this work.

| **Name** | **Characteristics and use** | **Source** |
| --- | --- | --- |
| *S. cerevisiae* (–) | BY4741 pRS413 + pRS416; Negative control for pulcherriminic production and target strain for fungal growth inhibition | This work |
| MpPul1Pul2_LC | BY4741 pPUL1Mp_LC + pPUL2Mp_LC; pulcherriminic acid production in low copy plasmids with *PUL1* and *PUL2* genes from yAMV511 | This work |
| MpPul1Pul2_HC | BY4741 pPUL1Mp_HC + pPUL2Mp_HC; pulcherriminic acid production in high copy plasmids with *PUL1* and *PUL2* genes from yAMV511 | This work |
| KlPul1Pul2_LC | BY4741 pPUL1Kl_LC + pPUL2Kl_LC; pulcherriminic acid production in low copy plasmids with genes from *K. lactis* | This work |
| KlPul1Pul2_HC | BY4741 pPUL1Kl_HC + pPUL2Kl_HC; pulcherriminic acid production in high copy plasmids with genes from *K. lactis* | This work |
| MpPul1KlPul2 | BY4741 pPUL1Mp_LC + pPUL2Kl_LC; pulcherriminic acid production with low copy plasmids with *PUL1* from yAMV511 and *PUL2* from *K. lactis* | This work |
| KlPul1MpPul2 | BY4741 pPUL1Kl_LC + pPUL2Mp_LC; pulcherriminic acid production with low copy plasmids with PUL1 from *K. lactis* and PUL2 from yAMV511 | This work |
| *S. cerevisiae pul3∆* | BY4741 *pul3∆* empty plasmid 1 + empty plasmid 2; Target strain for fungal growth inhibition | This work |
| *pul3∆*_KlPul1Pul2 | BY4741 *pul3∆* pPUL1Kl + pPUL2Kl; pulcherriminic acid production with low copy plasmids with genes from *K. lactis* | This work |
| *pul3∆*_KlPul1Pul2Pul3 | BY4741 *pul3∆* pPUL1Kl + pPUL2Kl_PUL3Kl; pulcherriminic acid production with low copy plasmids with genes from *K. lactis* | This work |
| *C. auris* ATCC-MYA-5001 | Target strain for fungal growth inhibition | American Type Culture Collection |
| yAMV240 | Wild isolate - species identification; *PUL1* and *PUL2* sequencing | (Maciá Valero, Tabatabaeifar and Billerbeck 2025) |
| yAMV312 | Wild isolate - species identification; *PUL1* and *PUL2* sequencing | (Maciá Valero, Tabatabaeifar and Billerbeck 2025) |
| yAMV460 | Wild isolate - species identification; *PUL1* and *PUL2* sequencing | (Maciá Valero, Tabatabaeifar and Billerbeck 2025) |
| yAMV511 | Wild isolate - species identification; *PUL1* and *PUL2* sequencing; pulcherriminic acid production via LC–MS | (Maciá Valero, Tabatabaeifar and Billerbeck 2025) |
| yAMV636 | Wild isolate – species identification; *PUL1* and *PUL2* sequencing | (Maciá Valero, Tabatabaeifar and Billerbeck 2025) |
| yAMV642 | Wild isolate – species identification; *PUL1* and *PUL2* sequencing | (Maciá Valero, Tabatabaeifar and Billerbeck 2025) |
| yAMV32 | Wild isolate – species identification | (Maciá Valero, Tabatabaeifar and Billerbeck 2025) |
| yAMV41 | Wild isolate – species identification | (Maciá Valero, Tabatabaeifar and Billerbeck 2025) |
| yAMV99 | Wild isolate – species identification | (Maciá Valero, Tabatabaeifar and Billerbeck 2025) |
| yAMV160 | Wild isolate – species identification | (Maciá Valero, Tabatabaeifar and Billerbeck 2025) |
| yAMV174 | Wild isolate – species identification | (Maciá Valero, Tabatabaeifar and Billerbeck 2025) |
| yAMV186 | Wild isolate – species identification | (Maciá Valero, Tabatabaeifar and Billerbeck 2025) |
| yAMV204 | Wild isolate – species identification | (Maciá Valero, Tabatabaeifar and Billerbeck 2025) |
| yAMV215 | Wild isolate – species identification | (Maciá Valero, Tabatabaeifar and Billerbeck 2025) |
| yAMV217 | Wild isolate – species identification | (Maciá Valero, Tabatabaeifar and Billerbeck 2025) |
| yAMV233 | Wild isolate – species identification | (Maciá Valero, Tabatabaeifar and Billerbeck 2025) |
| yAMV286 | Wild isolate – species identification | (Maciá Valero, Tabatabaeifar and Billerbeck 2025) |
| yAMV322 | Wild isolate – species identification | (Maciá Valero, Tabatabaeifar and Billerbeck 2025) |
| yAMV346 | Wild isolate – species identification | (Maciá Valero, Tabatabaeifar and Billerbeck 2025) |
| yAMV360 | Wild isolate – species identification | (Maciá Valero, Tabatabaeifar and Billerbeck 2025) |
| yAMV380 | Wild isolate – species identification | (Maciá Valero, Tabatabaeifar and Billerbeck 2025) |
| yAMV420 | Wild isolate – species identification | (Maciá Valero, Tabatabaeifar and Billerbeck 2025) |
| yAMV564 | Wild isolate – species identification | (Maciá Valero, Tabatabaeifar and Billerbeck 2025) |
| yAMV610 | Wild isolate – species identification | (Maciá Valero, Tabatabaeifar and Billerbeck 2025) |
| yAMV623 | Wild isolate – species identification | (Maciá Valero, Tabatabaeifar and Billerbeck 2025) |
| yAMV660 | Wild isolate – species identification | (Maciá Valero, Tabatabaeifar and Billerbeck 2025) |
| yAMV669 | Wild isolate – species identification | (Maciá Valero, Tabatabaeifar and Billerbeck 2025) |
| yAMV692 | Wild isolate – species identification | (Maciá Valero, Tabatabaeifar and Billerbeck 2025) |
| yAMV702 | Wild isolate – species identification | (Maciá Valero, Tabatabaeifar and Billerbeck 2025) |
| yAMV721 | Wild isolate – species identification | (Maciá Valero, Tabatabaeifar and Billerbeck 2025) |

**Supplementary Table 2.** Oligonucleotides used in this study for species identification, sequencing of *PUL* cluster in wild yeast isolates and molecular cloning of *PUL* genes in a *S. cerevisiae* heterologous system.

| **Name** | **Description** | **Source** | **Sequence (5' → 3')** |
| --- | --- | --- | --- |
| Species identification | | | |
| ITS3 (fw) | ITS2 region from wild yeast isolates | (White *et al.* 1990) | GCATCGATGAAGAACGCAGC |
| ITS4 (rv) | ITS2 region from wild yeast isolates | (White *et al.* 1990) | TCCTCCGCTTATTGATATGC |
| NL1 (fw) | D1/D2 domain from wild yeast isolates | (O´Donnell 1993) | GCATATCAATAAGCGGAGGAAAAG |
| NL4 (rv) | D1/D2 domain from wild yeast isolates | (O´Donnell 1993) | GGTCCGTGTTTCAAGACGG |
| *PUL* cluster identification and sequencing | | | |
| PUL1fw | *PUL1* from wild yeast isolates | This work | AGAATACAGGTGGGCTCA |
| PUL1midfw | *PUL1* from wild yeast isolates | This work | ATGAAATGATATGCACCCACA |
| PUL1rv | *PUL* cluster from wild yeast isolates | This work | TTCTAGATAAAGAGAACACCCTGTT |
| PUL2midrv | *PUL2* from wild yeast isolates | This work | TTTATCGTTGGCCATGGTG |
| PUL2rv | *PUL2* from wild yeast isolates | This work | AATTGGTTTCCTAATCGGGA |
| PUL3fw | *PUL* cluster from wild yeast isolates | This work | TTACAGTTACATCACAGCCATAC |
| *Molecular cloning* | | | |
| SB618 | *PUL1* fw from *K. lactis NRRL Y–1140* | This work | GCATCGTCTCATCGGTCTCATATGTACCAACTGCTTTTCC |
| SB619 | *PUL1* rv from *K. lactis* NRRL Y–1140 | This work | ATGCCGTCTCAGGTCTCAGGATTCAGATTACGAGAGCACCA |
| SB620 | *PUL2* fw from *K. lactis NRRL Y–1140* | This work | GCATCGTCTCATCGGTCTCATATGTTAGCTGATATATTAATCCCA |
| SB621 | *PUL2* rv from *K. lactis* NRRL Y–1140 | This work | ATGCCGTCTCAGGTCTCAGGATTCACAATGCAGTTAGTT |
| SB622 | *PUL3* fw from *K. lactis NRRL Y–1140* | This work | GCATCGTCTCATCGGTCTCATATGAAGCTAACAGATTCACAAA |
| SB623 | *PUL3* rv from *K. lactis* NRRL Y–1140 | This work | ATGCCGTCTCAGGTCTCAGGATCACATTTTGTTCCTCCTAAG |
| SB626 | *PUL3* fw (BsaI outcloning) | This work | GCATCGTCTCAGAGCGGTATCTTTAAACACGTTACTTG |
| SB627 | *PUL3* rv (BsaI outcloning) | This work | GCATCGTCTCAGCTCCTGCACAGGAATATA |
| SB628 | *PUL2* fw (BsaI outcloning) | This work | GCATCGTCTCAGAGATCAGTGTGGTGACCTG |
| SB629 | *PUL2* rv (BsaI outcloning) | This work | GCATCGTCTCATCTCTGTTTTTGATAGACAAGCTTTTGCCG |

**Supplementary Table 3.** DNA sequence of *PUL1/2* from yAMV511 ordered as gBlocks. The shaded part of the sequence indicates the overhangs necessary to clone the oligonucleotides into plasmids following the MoClo system into Type 1 plasmids (Lee *et al.* 2015).

| PUL1Mp | GCATCGTCTCATCGGTCTCATATGGTGACTGAATCAGGCGAGCTGTTCATAAGCCAGGCCTTCATAGACATGTGGATAGATAGCCTAGTTGGTTATCTACCCGGAGAAACGTGCAGACACGTTTCTCAATTTGTCCAGAATGAGTACGTGGGTACGCTGGGTCAATTATACGTCACCATAAGGGACTTGAGGCAGGCCATCAGCGCATTCGTGGACAGTGTTAATGGCGAAGAAAATAAAAATTTTTTGGCCTTTGATGCACATGTATCTGACTGTGGTTGCCATTTGCGTGCGTCCATGAGTATGGACTTGATTCAAAGATACAGGGGCAATAGGGAAGAGTTACTGAGTTTTCTAGGCTTGGTCGAAGCTTGCGATAGGGCTCTGGTCAGTACAAGTGCCTTAATGAAGGATATATGCACTGAGGCGAAAAGCTTGAAAGAATTACAACTACCTAAAAGTACCAAAGATCCTTTACTGTTTTTGAACGCAATCGGCTGGAAATTTGAATCTAACAACTTGAGTGAAATAAAATATATCTTCTACTGCTACGTGTTATCACAATTCAAGACGTATAGCTTCAGGAACAAACAAGATTCAGTCCACATTGATACAGACAAAGAATTTAAACAGAAGAATGAAATGATTTGTACTCACACCTGTCAGGGCAAGGGAAAGCTGGGAAATGGGTGTAGATATCTGAAGCACGCGAGGATTGGGAAGGCTGCGCTGAAACAATGGACACTTTGTTATCAGGAGAGGCTGTCCAAAATGTCCGTGGACTATCTTGCTAAGTCAGACTCAGAGTTGAAAGAGCTGGTAGAGACAAGCCGTAAAGAATCCCATAAGTCTGTCGCAGCAGTACCGTCTTATGTCCAATTTAAAATATCCGAGCGTCTGTGGGCGAGTAATCAATTCCCATTTCTTCTTAGTATGAGAGTTTTTGTCGATGAGGGGCACGACGATATATACGCCCGTGCCTTCGTCGGTCGTGATCTTAAATGGAACATCCAATTCGTAACATCAGACGTCTTAGAGGATACGCCACACATTATCGTGGCAGGGCATTGTCGTGTACCCCACGGCTACAATGACATAAACAAGTTAGACCTAGCTAACTTGTCACTTGATGCTCATCAAAATATGAGGAGTTTTTGGTATTCATTTATGTCACAACACAAACAGTACCCCTTTGACACCGCCCTAGGCTGTGATGATGACCTTCAAAATGTTTTACCTGCCCATGAATTTAAGGATTACATGAAGTTTAAGTATGCCGGCATAAGGGCATTTCGTGATATGGAATTCACACCAAAGCACATTTTTGTTGAATATCCAAGTATCGTGTTCAGTAAGCAGAGAATGTTGGCTGGGAAACAGGGCGTCCTATTCATTTAGATCCTGAGACCTGAGACGGCAT |
| --- | --- |
| PUL2Mp | GCATCGTCTCATCGGTCTCATATGCTAACCATACTATCCCTTCCGGTCTTTTGGGTACTTGTAGTTAGTTTGTGTCTAGTCAGTTCCAAAAGTAAACTTAGGGCCTTTCAAAAGCAACCACCATTTAAGAAGATACCCAGATCCCATCGTAACGTAGAGGGAAAGAAAATTACACAGGGTCCGGAGATCTCTGAGAAAAACAAAAATCAGTACGGCAGCATATATTCACATCGTGATGGGTTTAGATATGAGGTCGTTCTAACAACGCCTAGCCAATTAAAACAATACTACAGCTCTCATACGAAAGATCACAAGAAACTGGATAGCTTTGGAGCAGGGCAGTATCTTGTCGCGCTTCTGGGGGAATGTCTTGGGTTCCAAAACGGGGAGAGTTGGACCAGAATGAGAAAGAGTTTTAACTTGTTTTTCACCCATACCTTAGCCGCTAAGACACTTCCTGCAATGATAGCGTTCATCGACGGATGGATTTCTGAGCATGACAGCCAGAATGAATTCAGTGTAGACGCCTTCGACTTCGTTGCAACCGTACCATTTACTTGCATTGCCAAGTATCTGTACGGAAACGAGCAGTGCAGCGGGCGTGTCCTGACTGAGCTTAAGAACCTTGTACCTTTGCACAGTGAACTTATGACTCACGCCTTTACCACTTTCTGGGGTCGTTTTCGTATCTACCAATACTTCCCTTTTCAGAGGATGAAGGATTTGAAGTATTTTCAAGATTCTTTCAAGTCACTTAGCCTAGCAATGGTCGAGTCAGCCCGTGACGCCGAGAACCCAACTGTTGCTTCCGAATTGTACAAGTTAGTCGAAAGCCGTGATTTGACGCTTGATAATTGGATACAATCCCTAGACGAGATTCTGTTCGCCAATATCGATGTTACTGCTACGATAATGTCTTGGTCATTGGTTGAGATGGGTCGTAACAAACACGAGCAAGCCCGTCTGAGGCTTGAGGTACTGGAGAACCTTCAGTCAGTAGACGAATATTGCAAAAGAACAGACACTGTTCTACATAGAGTCTTACTAGAGATCCTTAGGCTTCATCCACTATTGTGGTATGGTTTCCCCGAACAAAGTAGCAGTGCAATGGTTATCGATGGTCACAAGATCGAGGCTAACACTCCCATTGTGGTTGATCAGTACCAATTAAATTACAAGAGCCCATTATGGAACCCAGCGGATAAGAGCACGGACTATGGAGCAACTTTCGATTCCAACCGTTTTCTAGGACTTAATAATCGTGATATATTGATGTCCTCTGTAACGTTTGGGAGCGGGCCTCGTAGGTGCTTAGGGAAAAACTTCGCCGAAGTTCTGATCAAGACGGAAGTGGCGAAGGTGTTGAGCACATTCGAGGTCGCGCTTGAAGGGGAGTTAAAGGTAGCTGCCGACACTTTCGTTGTAAGACCCGATGCGCAAATAAAACTTACAAGGTTGATATAGATCCTGAGACCTGAGACGGCAT |

**Supplementary Table 4.** Plasmids used in this work.

| **Name** | **Characteristics and use** | **Source** | **Link** |
| --- | --- | --- | --- |
| *Yeast Toolkit parts* | | | |
| pYTK001 | Entry vector | (Lee *et al.* 2015) | https://www.addgene.org/65108/ |
| pYTK002 | conLS (Type 1) | (Lee *et al.* 2015) | https://www.addgene.org/65109/ |
| pYTK009 | pTDH3 (Type 2) | (Lee *et al.* 2015) | https://www.addgene.org/65116/ |
| pYTK010 | pCCW12 (Type 2) | (Lee *et al.* 2015) | https://www.addgene.org/65117/ |
| pYTK014 | pTEF2 (Type 2) | (Lee *et al.* 2015) | https://www.addgene.org/65121/ |
| pYTK047 | GFPdropout (Type 234r) | (Lee *et al.* 2015) | https://www.addgene.org/65154/ |
| pYTK051 | tENO1 (Type 4) | (Lee *et al.* 2015) | https://www.addgene.org/65158/ |
| pYTK052 | tSSA1 (Type 4) | (Lee *et al.* 2015) | https://www.addgene.org/65159/ |
| pYTK053 | tADH1 (Type 4) | (Lee *et al.* 2015) | https://www.addgene.org/65160/ |
| pYTK072 | conRE (Type 5) | (Lee *et al.* 2015) | https://www.addgene.org/65179/ |
| pYTK074 | URA3 (Type 6) | (Lee *et al.* 2015) | https://www.addgene.org/65181/ |
| pYTK076 | HIS3 (Type 6) | (Lee *et al.* 2015) | https://www.addgene.org/65183/ |
| pYTK081 | CEN6/ARS4 (Type 7) | (Lee *et al.* 2015) | https://www.addgene.org/65188/ |
| pYTK082 | 2 micron (Type 7) | (Lee *et al.* 2015) | https://www.addgene.org/65189/ |
| pYTK083 | AmpR–ColE1 (Type 8) | (Lee *et al.* 2015) | https://www.addgene.org/65190/ |
| pYTK084 | KanR–ColE1 (Type 8) | (Lee *et al.* 2015) | https://www.addgene.org/65191/ |
| pYTK095 | AmpR–ColE1 (Type 678) | (Lee *et al.* 2015) | https://www.addgene.org/65202/ |
| *Plasmids constructed* | | | |
| EP1 | pRS413: Negative control | This work | https://benchling.com/s/seq–Plf3Ui11zvNvyrxJ6DQI?m=slm–YA7sS7zLQobwxtyupcys |
| EP2 | pRS416; Negative control | This work | https://benchling.com/s/seq–0pguFzdsGasAf9ZlqMNI?m=slm–t7AUiC0YXFMxlocq58Wg |
| pPUL1Mp_LC | *PUL1* from *Metschnikowia* sp yAMV511 in low–copy plasmid | This work | https://benchling.com/s/seq–K5mBajixUn3DECn9A49d?m=slm–zIEXfcUJtRJ8CUZod67b |
| pPUL1Mp_HC | *PUL1* from *Metschnikowia* sp yAMV511 in high–copy plasmid | This work | https://benchling.com/s/seq–9R5BzrKMgaEQSRCXty6n?m=slm–kKUjea3azkQxgJXaX999 |
| pPUL2Mp_LC | *PUL2* from *Metschnikowia* sp yAMV511 in low–copy plasmid | This work | https://benchling.com/s/seq–ugapG7UYTtP2vWY7zXKi?m=slm–nn702CTEFPOD526sznRy |
| pPUL2Mp_HC | *PUL2* from *Metschnikowia* sp yAMV511 in high–copy plasmid 1 | This work | https://benchling.com/s/seq–robtQMtdM2HmgyfiHxxq?m=slm–ZEeS1R5ThNWoNpCt2nDS |
| pPUL1Kl_LC | *PUL1* from *K. lactis* in low–copy plasmid | This work | https://benchling.com/s/seq–YMPq7KStg046NbcNMw7s?m=slm–41YHDDBQMoIAyzHrxUpL |
| pPUL1Kl_HC | *PUL1* from *K. lactis* in high–copy plasmid | This work | https://benchling.com/s/seq–Z0RXnM2FkzIDvUAUudoF?m=slm–dcr3tYWtOgBo08qIQ4Og |
| pPUL2Kl_LC | *PUL2* from *K. lactis* in low–copy plasmid | This work | https://benchling.com/s/seq–naWL6n0lxD4FCT24kOo3?m=slm–VLNdBIEJqf04W3lyNJWO |
| pPUL2Kl_HC | *PUL2* from *K. lactis* in high–copy plasmid | This work | https://benchling.com/s/seq–l5EEp7OnGp0kIp29KoS5?m=slm–iacKt48pHGCWRXCM7Mvv |
| pPUL2Kl_PUL3Kl | *PUL2/3* from *K. lactis* in low–copy plasmid | This work | https://benchling.com/s/seq–xSrSiOCXK64DQ1wn3ZlW?m=slm–bCDzkUfN6OYWU1ZXIFTQ |

**Supplementary Table 5.** Species identification of yeast isolates producing iron chelators. The table shows BLAST hits with highest sequence similarity. Isolates were linked to a given species when 97% sequence similarity was reached and to a given genus when >95% was reached from the hits retrieved. All isolates belong to the *Metschnikowia* clade.

| **yAMV #** | **OTU** | **Locus** | **Sequence (5' → 3')** |
| --- | --- | --- | --- |
| 32 | *M. pulcherrima* | D1/D2 | TCAGTAACGGCGAGTGAAGCGGCAAAAGCTCAAATTTGAAATCCCCCGGGAATTGTAATTTGAAGAGATTTGGGTCCGGCCGGCGGGGGTTAAGTCCACTGGAAAGTGGCGCCACAGAGGGTGACAGCCCCGTGAACCCCTTTAACGCCCTCATCCCAGATCTCCAAGAGTCGAGTTGTTTGGGAATGCAGCTCTAAGTGGGTGGTAAATTCCATCTAAAGCTAAATACCGGCGAGAGACCGATAGCGAACAAGTACAGTGATGGAAAGATGAAAAGCACTTTGAAAAGAGAGTGAAAAAGTACGTGAAATTGTTGAAAGGGAAGGGCTTGCAAGCAGACACTTAACTGGGCCAGCATCGGGGCGGCGGGAAACAAAACCACCGGGGAATGTACCTTTCGAGGATTATAACCCCGGTCTCTATTTCCTTGTTGCCCCGAGGCCTGCAATCTAAGGATGCTGGCGTAATGGTTGCAAGTCGCCCGTCT |
|  | *M. pulcherrima* | ITS2 | CTTGCAGTAACGTGAATCATTGAATCTTCTGAACGCACATCTGCGTCTCTCTCGGGGTATTCCCCAGGGCATGCGTGGGTGAGCGATATTTACTCTCAAACCTCCGGTTTGGTCCTGCTTCGGCCTAATATCAACGGCGCTAGAATAAGTTTTAGCCCCATTCTTTTTCCTCACCCTCGTAAGACTACCCGCTGAACTTAAGCATATCATAAAGCCGG |
| 240 | *M. pulcherrima* | D1/D2 | GAAGAGATTTGGGTCCGGCCGGCGGGGGTTAAGTCCACTGGAAAGTGGCGCCACAGAGGGTGACAGCCCCGTGAACCCCCTCAACGCCCTCATCCCAGATCTCCAAGAGTCGAGTTGTTTGGGAATGCAGCTCTAAGTGGGTGGTAAATTCCATCTAAAGCTAAATACCGGCGAGAGACCGATAGCGAACAAGTACAGTGATGGAAAGATGAAAAGCACTTTGAAAAGAGAGTGAAAAAGTACGTGAAATTGTTGAAAGGGAAGGGCTTGCAAGCAGACACTTAACTGGGCCAGCATCGGGGCGGCGGGAAACAAAACCACCGGGGAATGTACCTCTCGAGGATTATAACCCCGGTCTCAATTTCCTCGCCGCCCCGAGGCCTGCAATCTAAGGATGCTGGCGTAATGGTTGCAAGTCGCCC |
|  | *M. pulcherrima* | ITS2 | CCCCGGGGTATTCCCCAGGGCATGCGTGGGTGAGCGATATTTACTCTCAAACCTCTGGTTTGGTCCTGCTTCGGCCTAATATCAACGGCGCTAGAATAAGTTTTAGCCCCATCCTTTTTCCTCACCCTCGTAAGACTACCCGCTGAACTTAAGCATATCATAAAAGCGGAGG |
| 286 | *M. pulcherrima* | D1/D2 | AAATCCCCCGGGAATTGTAATTTGAAGAGATTTGGGTCCGGCCGGCAGGGGTTAAGTCCACTGGAAAGTGGCGCCACAGAGGGTGACAGCCCCGTGAACCCCCTCAACGCCCTCATCCCAGATCTCCAAGAGTCGAGTTGTTTGGGAATGCAGCTCTAAGTGGGTGGTAAATTCCATCTAAAGCTAAATACCGGCGAGAGACCGATAGCGAACAAGTACAGTGATGGAAAGATGAAAAGCACTTTGAAAAGAGAGTGAAAAAGTACGTGAAATTGTTGAAAGGGAAGGGCTTGCAAGCAGACACTTAACTGGGCCAGCATCGGGGCGGCGGGAAACAAAACCACCGGGGAATGTACCTTTCGAGGATTATAACCCCGGTCTCAATTTCCATGTTGCCCCGAGGCCTGCAATCTAAGGATGCTGGCGTAATGGTTGCAAGTCGCCCGT |
|  | *M. pulcherrima* | ITS2 | TTGAATCTTTGAACGCACATTGCGCCCCGGGGTATTCCCCAGGGCATGCGTGGGTGAGCGATATTTACTCTCAAACCTCCGGTTTGGTCCTGCTTCGGCCTAATATCAACGGCGCTAGAATAAGTTTTAGCCCCATCCTTTTTCCTCACCCTCGTAAGACTACCCGCTGAACTTAAGCATATCAATAAGCGGAGGAA |
| 312 | *M. pulcherrima* | D1/D2 | TCCCCCGGGAATTGTAATTTGAAGAGATTTGGGTCCGGCCGGCGGGGGTTAAGTCCACTGGAAAGTGGCGCCACAGAGGGTGACAGCCCCGTGAACCCCTTCAACGCCCTCATCCCAGATCTCCAAGAGTCGAGTTGTTTGGGAATGCAGCTCTAAGTGGGTGGTAAATTCCATCTAAAGCTAAATACCGGCGAGAGACCGATAGCGAACAAGTACAGTGATGGAAAGATGAAAAGCACTTTGAAAAGAGAGTGAAAAAGTACGTGAAATTGTTGAAAGGGAAGGGCTTGCAAGCAGACACTTAACTGGGCCAGCATCGGGGCGGCGGGGAGCAAAACCACCGGGGAATGTACCTTTCGAGGATTATAACCCCGGCCCTTACTCCCATACTGCCCCGAGGCCTGCATTCTAAGGATGCTGGCGTAATGGTTGCAAGTCGCCCGTC |
|  | *M. pulcherrima* | ITS2 | TTTGAATCTTTTGAACGCACTCTGCGCCCCGGGGTATTCCCAGGGCATGCGTGGGTGAGCGATATTTACTCTCAAACCTCCGGTTTTGGTCCTGCTTCGGCCTAATATCAACGGCGTCTAGAATAAGTTTTAGCCCCATTCTTCTTCCTCACCCTCGTAAGACTACCCGCTGAACTTAAGCATA |
| 420 | *M. pulcherrima* | D1/D2 | GTGAGCGGCAAAAGCTCAAATTTGAAATCCCCCGGGAATTGTAATTTGAAGAGATTTGGGTCCGGCCGGCAGGGGTTAAGTCCACTGGAAAGTGGCGCCACAGAGGGTGACAGCCCCGTGAACCCCTTTAACGCCCTCATCCCAGATCTCCAAGAGTCGAGTTGTTTGGGAATGCAGCTCTAAGTGGGTGGTAAATTCCATCTAAAGCTAAATACCGGCGAGAGACCGATAGCGAACAAGTACAGTGATGGAAAGATGAAAAGCACTTTGAAAAGAGAGTGAAAAAGTACGTGAAATTGTTGAAAGGGAAGGGCTTGCAAGCAGACACTTAACTGGGCCAGCATCGGGGCGGCGGGAAACAAAACCACCGGGGAATGTACCTTTCGAGGATTATACCCCCGGTCTCTATTTCCTTGCTGCCCCGAGGCCTGCAATCTAAGGATGCTGGCGTAATGGTTGCAAGTCGCC |
|  | *M. pulcherrima* | ITS2 | TTGAACTTTGCAGTAACGTTGAATCATTTGAAATCCTTTTGAACGCACATCTGCGCCCCGGGGTATTCCCCAGGGCATGCGTGGGTGAGCGATATTTACTCTCAAACCTCCGGTTTGGTCCTGCTTCGGCCTAATATCAACGGCGCTAGAATAAGTTTTAGCCCCAGCCTTTTTCCTCACCCTCGTAAGACTACCCGCTGAACTTAAGCATAT |
| 460 | *M. pulcherrima* | D1/D2 | AATTTGAAGAGATTTGGGTCCGGCCGGCGGGGGTTAAGTCCACTGGAAAGTGGCGCCACAGAGGGTGACAGCCCCGTGAACCCCTTCAACGCCCTCATCCCAGATCTCCAAGAGTCGAGTTGTTTGGGAATGCAGCTCTAAGTGGGTGGTAAATTCCATCTAAAGCTAAATACCGGCGAGAGACCGATAGCGAACAAGTACAGTGATGGAAAGATGAAAAGCACTTTGAAAAGAGAGTGAAAAAGTACGTGAAATTGTTGAAAGGGAAGGGCTTGCAAGCAGACACTTAACTGGGCCAGCATCGGGGCGGCGGGAAACAAAACCACCGGGGAATGTACCTTTCGAGGATTATAACCCCGGTCTCTATTTCCTCGCCACCCCGAGGCCTGCAATCTAAGGATGCTGGCGTAATGGTTGCAAGTCGCCCGTTA |
|  | *M. pulcherrima* | ITS2 | AGCGATATTTACTCTCAAACCTCCGGTTTGGTCCTGCTTCGGCCTAATATCAACGGCGCTAGAA |
| 511 | *Metschnikowia* sp | D1/D2 | GTACGGCGAGTGAAGCGGCAAAAGCTCCAAATTTGAAATTCCCCCCGGGAATTGTAATTTGAAGAAGATTTGGGTCCGGCCGGCGGGGGTTAAGTCCACTGGAAAGTGGCGCCACAGAGGGTGACAGCCCCGTGAACCCCTTCAACGCCCTCATCCCAGATCTCCAAGAGTCGAGTTGTTTGGGAATGCAGCTCTAAGTGGGTGGTAAATTCCATCTAAAGCTAAATACCGGCGAGAGACCGATAGCGAACAAGTACAGTGATGGAAAGATGAAAAGCACTTTGAAAAGAGAGTGAAAAAGTACGTGAAATTGTTGAAAGGGAAGGGCTTGCAAGCAGACACTTAACTGGGCCAGCATCGGGGCGGCGGGAAACAAAACCACCGGGGAATGTACCTTTCGAGGATTATAACCCCGGTCTCAATTTCCTTGCCGCCCCGAGGCCTGCAATCTAAGGATGCTGGCGTAATGGTTGCAAGTCGC |
|  | *Metschnikowia* sp | ITS2 | TGATGCGATATTTACTCTCAAACCTCTCGGTTTTCGGTCCTTCGCTTTCGGCCTAATATCAACGGCGCTCGAATAAGTTTTAGCTCATTCTTTTTCCTCACCCTCGTAAGATACCCGCTGAACTTAAGCATACGAATCGAAGGCA |
| 636 | *M. pulcherrima* | D1/D2 | CGGCGAGTGAGCGGCAAAAGCTCAAATTTGAAATCCCCCGGGAATTGTAATTTGAAGAGATTTGGGTCCGGCCGGCGGGGGTTAAGTCCACTGGAAAGTGGCGCCACAGAGGGTGACAGCCCCGTGAACCCCTTTAACGCCCTCATCCCAGATCTCCAAGAGTCGAGTTGTTTGGGAATGCAGCTCTAAGTGGGTGGTAAATTCCATCTAAAGCTAAATACCGGCGAGAGACCGATAGCGAACAAGTACAGTGATGGAAAGATGAAAAGCACTTTGAAAAGAGAGTGAAAAAGTACGTGAAATTGTTGAAAGGGAAGGGCTTGCAAGCAGACACTTAACTGGGCCAGCATCGGGGCGGCGGGAAACAAAACCACCGGGGAATGTACCTTTCGAGGATTATAACCCCGGTCCTTACTCCCTTGCTGCCCCGAGGCCTGCAATCTAAGGATGCTGGCGTAATGGTTGCAAGTCGCCCGT |
|  | *M. pulcherrima* | ITS2 | GCGTGGGTGAGCGATATTTACTCTCAAACCTCTGGTTTGGTCCTGCTTCGGCCTAATATCAACGGCGCTGAATAAG |
| 642 | *M. pulcherrima* | D1/D2 | AAGCTCAAATTTGAAATCCCCCGGGAATTGTAATTTGAAGAGATTTGGGTCCGGCCGGCGGGGGTTAAGTCCACTGGAAAGTGGCGCCACAGAGGGTGACAGCCCCGTGAACCCCTTTAACGCCCTCATCCCAGATCTCCAAGAGTCGAGTTGTTTGGGAATGCAGCTCTAAGTGGGTGGTAAATTCCATCTAAAGCTAAATACCGGCGAGAGACCGATAGCGAACAAGTACAGTGATGGAAAGATGAAAAGCACTTTGAAAAGAGAGTGAAAAAGTACGTGAAATTGTTGAAAGGGAAGGGCTTGCAAGCAGACACTTAACTGGGCCAGCATCGGGGCGGCGGGAAACAAAACCACCGGGGAATGTACCTTTCGAGGATTATAACCCCGG |
|  | *M. pulcherrima* | ITS2 | CACATTGCGCCCCGGGGTATTCCCCAGGGCATGCGTGGGTGAGCGATATTTACTCTCAAACCTCCGGTTTGGTCCTGCTTCGGCCTAATATCAACGGCGCTAGAATAAGTTTTAGCCCCAGCCTTTTTCCTCACCCTCGCTAAGAGCTACCTCGCTGTAACTCT |
| 721 | *M. pulcherrima* | D1/D2 | TTTGAAATCCCCCGGGAATTGTAATTTGAAGAGATTTGGGTCCGGCCGGCGGGGGTTAAGTCCACTGGAAAGTGGCGCCACAGAGGGTGACAGCCCCGTGAACCCCTTTAACGCCCTCATCCCAGATCTCCAAGAGTCGAGTTGTTTGGGAATGCAGCTCTAGTGGGTGGTAAATTCCATCTAAAGCTAAATACCGGCGAGAGACCGATAGCGAACAAGTACAGTGATGGAAAGATGAAAAGCACTTTGAAAAGAGAGTGAAAAAGTACGTGAAATTGTTGAAAGGGAAGGGCTTGCAAGCAGACACTTAACTGGGCCAGCATCGGGGCGGCGGGAAACAAAACCACCGGGGAATGTACCTTTCGAGGATTATAACCCCGGTCTCAATTTCCTTGTTGCCCCGAGGCCTGCAATCTAAGGATGCTGGCGTAATGGTTGCAAGTCGCCCGTCTG |
|  | *M. pulcherrima* | ITS2 | CGTGAATCATTGAATCTTTGAACGCACATTGCGCCCCGGGGTATTCTCTCAGGGCATGCGTGGGTGAGCGATATTTACTCTCAAACCTCCGGTTTGGTCCTGCTTCGGCCTAATATCAACGGCGCTAGAATAAGTTTTAGCCCCAGCCTTTCCTCCTCACCCTCGTAAGACTACCCGCTGAACTATAAGCATA |
| 41 | *M. pulcherrima* | ITS2 | TGAGCGATATTTACTCTCAAACCTCCGGTTTGGTCCTGCTTCGGCCTAATATCAACGGCGCTAGAATAAGTTTTAGCCCCATCCTTTTTCCTCACCCTCGTAAGACTACCCGCTGAACTTAAGCATATCATAAAGCGGAGGAGAA |
| 99 | *Metschnikowia* sp | ITS2 | GGTGAGCGATATTTACTCTCAAACCTCCGGTTTGGTCCTGCTTCGGCCTAATATCAACGGCGCTAGAATAAGTTTTAGCCCCAGCCTTTTTCCTCACCCTCGTAAGACTACCCGCTGAACTTAAGCATATCATAGAAGCGG |
| 160 | *M. pulcherrima* | ITS2 | TGGGTGAGCGATATTTACTCTCAAACCTCCGGTTTGGTCCTGCTTCGGCCTAATATCAACGGCGCTAGAATAAGTTTTAGCCCCAGCCTTTTTCCTCACCCTCGTAAGACTACCCGCTGAACTTAAGCATATCA |
| 174 | *M. pulcherrima* | ITS2 | TTTACTCTCAAACCTCCGGTTTGGTCCTGCTTCGGCCTAATATCAACGGCGCTAGAATAAGTTTTAGCCCCATTCTTCTTCCTCACCCTCGTAAGACTACCCGCTGAACTTAAGCATATCA |
| 186 | *M. pulcherrima* | ITS2 | CATGCGTGGGTGAGCGATATTTACTCTCAAACCTCCGGTTTGGTCCTGCTTCGGCCTAATATCAACGGCGCTAGAATAAGTTTTAGCCCCATTCTTTTTCCTCACCCTCGTAAGACTACCCGCTGAACTTAAGCATATCAGTAAA |
| 204 | *Metschnikowia* sp | ITS2 | AGCGATATTTACTCTCAAACCTCCGGTTTGGTCCTGCTTCGGCCTAATATCAACGGCGCTAGAATAAGTTTTAGCCCCAGCCTTTTTCCTCACCCTCGTAAGACTACCCGCTGAACTTAAGCATATCATAAAGCGGAGGA |
| 215 | *M. pulcherrima* | ITS2 | TGAATCTTTGAACGCACATTGCGCCCCGGGGTATTCCCCAGGGCATGCGTGGGTGAGCGATATTTACTCTCAAACCTCCGGTTTGGTCCTGCTTCGGCCTAATATCAACGGCGCTAGAATAAGTTTTAGCCCCATTCTTTTTCCTCACCCTCGTAAGACTACCCGCTGAACTTAAGCATATCA |
| 217 | *M. pulcherrima* | ITS2 | TGAGCGATATTTACTCTCAAACCTCCGGTTTGGTCCTGCTTCGGCCTAATATCAACGGCGCTAGAATAAGTTTTAGCCCCATTCTTTTTCCTCACCCTCGTAAGACTACCCGCTGAACTTAAGCATATCATAAAGCGGAG |
| 233 | *M. pulcherrima* | ITS2 | GGCATGCGTGGGTGAGCGATATTTACTCTCAAACCTCCGGTTTGGTCCTGCTTCGGCCTAATATCAACGGCGCTAGAATAAGTTTTAGCCCCATTCTTTTTCCTCACCCTCGTAAGACTACCCGCTGAACTTAAGCATATCATAAAGCGGAGG |
| 322 | *M. pulcherrima* | ITS2 | CGCCCCGGGGTATTCCCCAGGGCATGCGTGGGTGAGCGATATTTACTCTCAAACCTCCGGTTTGGTCCTGCTTCGGCCTAATATCAACGGCGCTAGAATAAGTTTTAGCCCCATCCTTCTTCCTCACCCTCGTAAGACTACCCGCTGAACTTAAGCATATC |
| 346 | *M. pulcherrima* | ITS2 | ATGCGTGGGTGAGCGATATTTACTCTCAAACCTCCGGTTTGGTCCTGCTTCGGCCTAATATCAACGGCGCTAGAATAAGTTTTAGCCCCATTCTTTTTCCTCACCCTCGTAAGACTACCCGCTGAACTTAAGCATATCATAAAAGCGGAGG |
| 360 | *M. pulcherrima* | ITS2 | GCGATATTTACTCTCAAACCTCTGGTTTGGTCCTGCTTCGGCCTAATATCAACGGCGCTAGAATAAGTTTTAGCCCCAGCCTTTTTCCTCACCCTCGTAAGAGTACCCGCTGAACTTAAGCATATC |
| 380 | *M. pulcherrima* | ITS2 | AGCGATATTTACTCTCAAACCTCCGGTTTGGTCCTGCTTCGGCCTAATATCAACGGCGCTAGAATAAGTTTTAGCCCCATCCTTTTTCCTCACCCTCGTAAGACTACCCGCTGAACTTAAGCATATCAT |
| 564 | *M. pulcherrima* | ITS2 | CATGCGTGGGTGAGCGATATTTACTCTCAAACCTCTGGTTTGGTCCTGCTTCGGCCTAATATCAACGGCGCTAGAATAAGTTTTAGCCCCAGCCTTTTTCCTCACCCTCGTAAGACTACCCGCTGAACTTAAGCATATC |
| 610 | *M. pulcherrima* | ITS2 | TTACTCTCAAACCTCCGGTTTGGTCCTGCTTCGGCCTAATATCAACGGCGCTAGAATAAGTTTTAGCCCCATTCTTTTTCCTCACCCTCGTAAGACTACCCGCTGAACTTAAGCATATCA |
| 623 | *M. pulcherrima* | ITS2 | CCCCAGGGCATGCGTGGGTGAGCGATATTTACTCTCAAACCTCCGGTTTGGTCCTGCTTCGGCCTAATATCAACGGCGCTAGAATAAGTTTTAGCCCCATCCTTTTTCCTCACCCTCGTAAGACTACCCGCTGAACTTAAGCATATCATAAAGCGGAGGA |
| 660 | *M. pulcherrima* | ITS2 | CCCCAGGGCATGCGTGGGTGAGCGATATTTACTCTCAAACCTCCGGTTTGGTCCTGCTTCGGCCTAATATCAACGGCGCTAGAATAAGTTTTAGCCCCAGCCTTTTTCCTCACCCTCGTAAGACTACCCGCTGAACTTAAGCATATA |
| 669 | *M. pulcherrima* | ITS2 | GGGTGAGCGATATTTACTCTCAAACCTCCGGTTTGGTCCTGCTTCGGCCTAATATCAACGGCGCTAGAATAAGTTTTAGCCCCATCCTTTTTCCTCACCCTCGTAAGACTACCCGCTGAACTTAAGCATAC |
| 692 | *M. pulcherrima* | ITS2 | AGCGATATTTACTCTCAAACCTCCGGTTTGGTCCTGCTTCGGCCTAATATCAACGGCGCTAGAATAAGTTTTAGCCCCATCCTTTTTCCTCACCCTCGTAAGACTACCCGCTGAACTTAAGCATATCAT |
| 702 | *M. pulcherrima* | ITS2 | AGCGATATTTACTCTCAAACCTCCGGTTTGGTCCTGCTTCGGCCTAATATCAACGGCGCTAGAATAAGTTTTAGCCCCAGTCTTTTTCCTCACCCTCGTAAGACTACCCGCTGAACTTAAGCATATCAT |

**Supplementary Table 6.** Pul1–4 primary sequences from genome–available *K. lactis, C. auris* and *Metschnikowia* species reported to produce iron chelators and Pul1/2 *Metschnikowia* isolates from this study. The CTG codons from yeasts that belong to the CTG–clade have been translated to serine instead of leucine. Loci with two ORFs have been included as *a* and *b*.

| **Pul1** | |
| --- | --- |
| yAMV240 | MVTESGELFISQAFIDMWIDSLVGYLPGETCRHVSQFVQNEYVGTLGQLYVTIRDLRQAISAFVDSVNGEENKNFLAFDAHVSDCGCHLRASMSMDLIQRYRGNREELLSFLGLVDACDNALVSTSALMKDICTEAKSLKELQLPKSTKDPLLFLNAIGWKFESDNLSEIKYIFYCYVLSQFKTYSFRNK  QDSVHIDTDKEFKQKNEMICTHTCQGKGKLGNGCRYLKHARIGKAALKQWTLCYQERLSKMSVDYLAKSDSELKELVENSRKESHKSVAAVPSYVQFKISERLWAFNQFPFLLSMRVFVDENHEDIYARAFVGRDLKWNIQFVTSDVLEDTPHIIVAGHCRVPHGYNDTNKLNLANLSLDAHQNMRSFWYSFMSQHKQYPFDTALGCDDDLQNVLPAHEFKDYMKFKYAGIRAFRDMEFTPKHIFVEYPSVVFSKQRMLAGKQGVLFI |
| yAMV312 | MVTESGELFISQAFIDMWIDSLVGYLPGETCRHVSQFVQNEYVGTLGQLYVTIRDLRQAISAFVDSVNGEENKNFLAFDAHVSDCGCHLRASMSMDLIQRYRGNREELLSFLGLVEACDKALVSTSVLMKDICTEAKSLKELQLPKSTKDPLLFLNAIGWKFESNNLSEIKYIFYCYVLSQFKTYSFRNKQDSVHIDTDKEFKQKNEMICTHTCQGKGKLGNGCRYLKHARIGKAALKQWTLCYQERLSKMSVVYLAKSDSELKELVENSRKESHKSVAAVPSYVQFKISERLWASNQFPFLLSMRVFVDEGHEDIYARAFVGRDLKWNIQFVTSDVLEDTPHIIVAGHCRVPHGYNDINTLNLANLSLDAHQNMRSFWYSFMSQHKQYPFDTALGCDDDLQNVLPAHEFKDYMKFKYAGIRAFKDMEFTPKHIFVEYPSVVFSKQRMLAGKQGVLFI |
| yAMV460 | MVTESGELFISQAFIDMWIDSLVGYLPGETCRHVSQFVQNEYVGTLGQLYVTIRDLRQAISAFVDSVNGEENKNFLAFDAHVSDCGCHLRASMSMDLIQRYRGNREELLSFLGLVEACDNALVSTSALMKDICTEAKSLKELQLPKSTKDPLLFLNAIGWKFESNNLSEIKYIFYCYVLSQFKTYSFRNKQDSVHIDTDKEFKQKNEMICTHTCQGKGKLGNGCRYLKHARIGKAALKQWTLCYQERLSKMSVDFLAKSDSELKELVENSRKESHKSVAAVPSYVQFKISERLWASNQFPFLLSMRVFVDEGHEDIYARAFVGRDLKWNIQFVTSDVLEDTPHIIVAGHCRVPHGYNDTNKLNLANLSLDAHQNMRSFWYSFMSQHKQYPFDTALGCDDDLQNVLPAHEFKDYMKFKYAGIRAFRDMEFTPKHIFVEYPSVVFSKQRMLAGKQGVLFI |
| yAMV511 | MVTESGELFISQAFIDMWIDSLVGYLPGETCRHVSQFVQNEYVGTLGQLYVTIRDLRQAISAFVDSVNGEENKNFLAFDAHVSDCGCHLRASMSMDLIQRYRGNREELLSFLGLVEACDRALVSTSALMKDICTEAKSLKELQLPKSTKDPLLFLNAIGWKFESNNLSEIKYIFYCYVLSQFKTYSFRNKQDSVHIDTDKEFKQKNEMICTHTCQGKGKLGNGCRYLKHARIGKAALKQWTLCYQERLSKMSVDYLAKSDSELKELVETSRKESHKSVAAVPSYVQFKISERLWASNQFPFLLSMRVFVDEGHDDIYARAFVGRDLKWNIQFVTSDVLEDTPHIIVAGHCRVPHGYNDINKLDLANLSLDAHQNMRSFWYSFMSQHKQYPFDTALGCDDDLQNVLPAHEFKDYMKFKYAGIRAFRDMEFTPKHIFVEYPSIVFSKQRMLAGKQGVLFI |
| yAMV636 | MVTESGELFISQAFIDMWIDSLVGYLPGETCRHVSQFVQNEYVGTLGQLYVTIRDLRQAISAFVDSVNGEENKNFLAFDAHVSDCGCHLRASMSMDLIQRYRGNREELLSFLGLVDACDNALVSTSALMKDICTEAKSLKELQLPKSTKDPLLFLSAIGWKFESNNLSEIKYIFYCYVLSQFKTYSFRNKQDSVHIDTDKEFKQKNEMICTHTCQGKGKLGNGCRYLKHARIGKAALKQWTLCYQERLSKMSVDYLAKSDSELKELVETSRKESHKSVAAVPSYVQFKISERLWAFNQFPFLLSMRVFVDEGHEDIYARAFVGRDLKWNIQFVTSDVLEDTPHIIVAGHCRVPHGYNDTNKLNLANLSLDAHQNMRSFWYSFMSQHKQYPFDTALGCDDDLQNVLPAHEFKDYMKFKYAGIRAFRDMEFTPKHIFVEYPSIVFSKQRMLAGKQGVLFI |
| yAMV642 | MVTESGELFISQAFIDMWIDSLVGYLPGETCRHVSQFVQNEYVGTLGQLYVTIRDLRQAISAFVDSVNGEENKNFLAFDAHVSDCGCHLRASMSMDLIQRYRGNREELLSFLGLVEACDNALVSTSALMKDICTEAKSLKELQLPKSTKDPLLFLNAIGWKFESNNLSEIKYIFYCYVLSQFKTYSFRNKQDSVHIDTDKEFKQKNEMICTHTCQGKGKLGNGCRYLKHARIGKAALKQWTLCYQERLSKMSVDYLAKSDSELKELVENSRKESHKSVAAVPSYVQFKISERLWASNQFPFLLSMRVFVDEGHEDIYARAFVGRDLKWNIQFVTSDVLEDTPHIIVAGHCRVPHGYNDTNKLNLANLSLDAHQNMRSFWYSFMSQHKQYPFDTALGCDDDLQNVLPAHEFKDYMKFKYAGIRAFRDMEFTPKHIFVEYPSVVFSKQRMLAGKQGVLFI |
| *M. pulcherrima* APC1.2 | MVTESGELFISQAFIDMWIDSLVGYLPGETCRHVSQFVQNEYVGTLGQLYVTIRDLRQAISAFVDSVNGEENKNFLAFDAHVSDCGCHLRASMSMDLIQRYRGNRKELLSFLGLVDACDNALVSTSALMKDICTEAKSLKELQLPKSTKDPLLFLNAIGWKFESNNLSEIKYIFYCYVLSQFKTYSFRNKQDSVHIDTDKEFKQKNEMICTHTCQGKGKLGNGCRYLKHARIGKAALKQWTLCYQERLSKMSVDYLAKSDSELKELVENSRKESHKSVAAVPSYVQFKISERLWAFNQFPFLLSMRVFVDEGHEDIYARAFVGRDLKWNIQFVASDVLEDTPHIIVAGHCRVPHGYNDTNKLNLANLSLDAHQNMRSFWYSFMSQHKQYPFDTALGCDDDLQNVLPAHEFKDYMKFKSAGIRAFKDMEFTPKHIFVEYPSVVFSKQRMLAGKQGVLFI |
| *M. pulcherrima* AP47 | MVTESGELFISQAFIDMWIDSLVGYLPGETCRHVSQFVQNEYVGTLGQLYVTIRDLRQAISAFVDSVNGEENKNFLAFDAHVSDCGCHLRASMSMDLIQRYRGNREELLSFLGLVEACDKALVSTSVLMKDICTEAKSLKELQLPKSTKDPLLFLNAIGWKFESNNLSEIKYIFYCYVLSQFKTYSFRNKQDSVHIDTDKEFKQKNEMICTHTCQGKGKLGNGCRYLKHARIGKAALKQWTLCYQERLSKMSVDYLAKSDSELKELVENSRKVSHKSVAAVPSYVQFKISERLWASNQFPFLLSMRVFVDESHEDIYARAFVGRDLKWNIQFVTSDVLEDTPHIIVAGHCRVPHGYNDTNKLNLANLSLDAHQNMRSFWYSFMSQHKQYPFDTALGCDDDLQTVLPAHEFKDYMKFKYAGIRAFRDMEFTPKHIFVEYPSVVFSKQRMLAGKQGVLFI |
| *M. pulcherrima* KIOM G15050 (*a*) | MVTESGELFISQAFIDMWIDSLVGYLPGETCRHVSQFVQNEYVGTLGQLYVTIRDLRQAISAFVDSVNGEENKNFLAFDAHVSDCGCHLRASMSMDLIQRYRGNREEILSFLGLVEACDKALVSTSALMKDICTEAKSLKELQLPKSTKDPLLFLNAIGWEI |
| *M. pulcherrima* KIOM G15050 (*b*) | MHPHLPRKGKLGNGCRYLKHARIGKAALKQWTLCYQERLSKMSVDYLAKSDSELKELVENSRKESHKSVAAVPSYVQFKISERLWASNQFPFLLSMRVFVDEGHKDIYARAFVGRDLKWNIKFVTSDVLEDTPHIIVAGHCRVPHGYNDINKFNLANLSLDAHQNMRSFWYSFMSQHKQYPFDTALGCDDDLQNVLPAHEFKDYMKFKYAGIRAFRDMEFTPKHIFVEYPSVVFSKQRMLAGKQGVLFI |
| *M. rubicola* CBS 15344 | MVTESGELFISQAFIDMWIDSLVGYLPGETCRHLSQFVQNEYVGTLGQLYVTIRDLRSAISAFVDSVNGEENKNFLAFDAHVSDCGCHLRASMSMDLIQRYRGNREEILSFLGLVEACDKALVSTSALMKDICTEAKSLKELQLPKSTKDPLLFLNAIGWKFESDNLSEIKYIFYCYVLSQFKTYSFRNKQDSVHIDTDKEFKQKNEMICTHTCQGKGKLGNGCRYLKHARIGKAALKQWTLCYQERLSKMSVDYLAKSDSELKELVENSRKESHKSVAAVPSYVQFKISERLWAFNQFPFLLSMRVFVDEGHEDIYARAFVGRDLKWNIQFVTSDVLEDTPHIIVAGHCRVPHGYNDINKLNLANLSLDAHQNMRSFWYSFMSQHKQYPFDTALGCDDDLQNVLPAHEFKDYMKFKYAGIRAFRDMEFTPKHIFVEYPSIVFSKQRMLAGKQGVLFI |
| *M. chrysoperlae* NRRL Y–27615 | MVTESGELFISHPFTDMWIDSLLGYLPGETCRHVSQFVQNEYVGTLGQLYVTIMDLRQAVSAFVDSVNGEENKNFLAFDAHVSDCGCHLRASMSMDLIQRYRKNKKELILFRGLVDACDNALVSTSALMKDICTEAKSLKELQLPKSTKDPLLFLNAIGWKFESKNLSEIKYIFYCYVLSQFKTYSFRNKQDSVHIDTDMEFKQKNEMICTHTCQGKGKLGNGCRYLKHARIGKAALKQWTLCYQERLSKMSVDYLAKCDSELRELVENSRKESHKSVAAVPSYVQFKISERLWASNEFPFLLSMRVFVDEGHEDIYARAFVGRDLRWEIRSVTNQELKDTPHIVVAGHCHVPQGCDDISKLDLETFSLDMHQNMRSFWYSFMSQHKQYPFDTPLGCDDELQDVLPAHEFEDYMKFKYTGIRAFGDIEFTPKHIFVEYPSVVFNKQRMLAGKQGVLFI |
| *C. auris* B11221 | MWIDTLLGLLPGDKYFQQTFKRNEYVGALAQLCVTIMDLKLASEAFLGSIEGKETFMPFDAHVSDCGCHLRAQLSMEKVFHYKKHPEKLEVFRKVIEVCEGSLLRASELSKALCWSEQSPLEFGLPKQTATRDDFKIAIEWVSDKFDGNSDIKYVYYCYTLSRFKRYNERNLKDSVEFDLDAASKCLNSLVYGFTCQGKGKLGEGCRYLKHAKIGKGFVKTQTTALQARLSQMSVEYLVSLVPELSNELKTYCVKSKKGIYAVPALLQYKVLERVWSARRTPLFLAIRFFDGDLSKFYYTFAMVAKDLQWAFHNESKRNEPHIAITFDCEISGLACLKDDDLSNVMCNLQPKMRETWYTFMTQHKQYPFELCGSLEQDLEAKIHSEVEVPKFRNTFHEPNGANLEPKHIFLDYPRAVTEKQASISGKAGTLLIN |
| *K. lactis* NRRL Y–1140 | MYQLLFQRLGVTLTAGNDKKTSIPSNQLVGHLIGLILLCDDLNEAFADFQALLQNGIAISSSDRGYLVFDAHVTDCGCHLRAQMIQDVFSYFKNHEVTKLYIFDAVAKKLNELKRHCISLIQTLCWENTSAQKLGFPKNIKSYEELLKLLQWDTADISSAIADSFPMNPNNADQNEKGLVWNVTKLEVVLEFLYCSHFLSKNKIYNKKENLDSVTIDFNNAFEKRQLLSNKFHCQGTGKLGVGCRYLKHAKQSKNSFISVVSNLQSRLALLSIAFLKSRCTLPCDIEALQKNSPRNVSAIPNFLHFLILEREWSQNETPILLAVRKLHEHEHCDLYFEARINPHTFEWTLQHKECCEFEHHTPYIVITALATGSSTTKTAQLLAWELMKAQKNFRQFWLTFMSQHRQYPFEIEHDEDQLLETQVSQDIFELYCQSKREDRNQILFDDSTSLLPKHIFTEYPSIFFNFQKNVCSKHGALVI |
| **Pul2** | |
| yAMV240 | MLTILSFPVFWVLVVSLCLVSSKSKLRAFQKQPPFKKIPRSHRNVEGKKITQGPEISEKNKNQYGSIYSHRDGFRYEVVLTTPSQLKQYYSSHTKDHKKLDSFGAGQYLVALLGECLGFQNGESWTRMRKSFNLFFTHTLAAKTLPAMIAFIDGWISEHDSQNEFSVDAFDFVATVPFTCIAKYLYGSEQCSGRVLTELKNLVPLHSELMTHAFTTFWGRFKIYQYFPFQRMKDLKFFQDSFKSLSLAMVESARDAENPTVASELYRLVESRDLTLDNWIQSLDEILFANIDVTATIMSWSLVEMGRNKHEQARLRLEVLENLHSMDEYCKRTDTVLHRVLLEILRLHPLLWYGFPEQSSSAMVIDGHKIEANTPIVVDQYQLNYKSPLWNPTDKSTDYGATFDSNRFLGLNNRDILMSSVTFGSGPRRCLGKNFAEVLIKTEIAKVLSTFEVALEGELKVAADTFVVRPDAQIKLTRLI |
| yAMV312 | MITILSFPVFWVLVVSLCLVSSKSKLRAFQKQPPFKKIPRSHRNVEGKKITQGPEISEKNKNQYGSIYSHRDGFRYEVVLTTPSQLKQYYSSHTKDHKKLDSFGAGQYLVALLGECLGFQNGESWTRMRKSFNLFFTHTLAAKTLPAMIAFIDGWISEHDSQDEFSVDAFDFVATVPFTCIAKYLYGNEQCSGRVLTELKNLVPLHSELMTHAFTTFWGRFKIYQYFPFQRMKDLKFFQDSFKSLSLAMVESARDAENLTVASELYKLVESRDLTLDNWIQSLDEILFANIDVTATIMSWSLVEMGRNKHEQARLRLEVLENLQSVDEYCKRTDTVLHRVLLEILRLHPLLWYGFPEQSSSAMVIDGHKIEANTPIVVDQYQLNYKSPLWNPADKSTDYGATFDSDRFLGLNNRDILMSSVTFGSGPRRCLGKNFAEVLIKTEIAKVLSTFEVALEGELKVAADTFVVRPDAQIKLTRLI |
| yAMV460 | MITILSLPVFWVLVVSLCLVSSKSKLRAFQKQPPFKKIPRSHRNVEGKKITQGPEISEKNKNQYGSIYSHRDGFRYEVVLTTPSQLKQYYSSHTKDHKKLDSFGAGQYLVALLGECLGFQNGESWTRMRKSFNLFFTHTLAAKTLPAMIAFIDGWISEHDSQNEFSVDAFDFVATVPFTCIAKYLYGNEQCSGRILTELKNLVPLHSELMTHAFTTFWGRFKIYQYFPFQRMKDLKFFQDSFKSLSLAMVESARDAENPTVASELYKLVESRDLTLDNWIQSLDEILFANIDVTATIMSWSLVEMGRNKHEQARLRLEVLENLQSVDEYCKRTDTVLHRVLLEILRLHPLLWYGFPEQSSSAMVIDGHKIEANTPIVVDQYQLNYKSPLWNPADKRTDYGATFDSDRFLGLNNRDILMSSVTFGSGPRRCLGKNFAEVLIKTEIAKVLFTFEVALEGELKVAADTFVVRPDAQIKLTRLI |
| yAMV511 | MLTILSLPVFWVLVVSLCLVSSKSKLRAFQKQPPFKKIPRSHRNVEGKKITQGPEISEKNKNQYGSIYSHRDGFRYEVVLTTPSQLKQYYSSHTKDHKKLDSFGAGQYLVALLGECLGFQNGESWTRMRKSFNLFFTHTLAAKTLPAMIAFIDGWISEHDSQNEFSVDAFDFVATVPFTCIAKYLYGNEQCSGRVLTELKNLVPLHSELMTHAFTTFWGRFRIYQYFPFQRMKDLKYFQDSFKSLSLAMVESARDAENPTVASELYKLVESRDLTLDNWIQSLDEILFANIDVTATIMSWSLVEMGRNKHEQARLRLEVLENLQSVDEYCKRTDTVLHRVLLEILRLHPLLWYGFPEQSSSAMVIDGHKIEANTPIVVDQYQLNYKSPLWNPADKSTDYGATFDSNRFLGLNNRDILMSSVTFGSGPRRCLGKNFAEVLIKTEVAKVLSTFEVALEGELKVAADTFVVRPDAQIKLTRLI |
| yAMV636 | MLTILSLPVFWVLVVSLCLVSSKSKLRAFQKQPPFKKIPRSHRNVEGKKITQGPEISEKNKNQYGSIYSHRDGFRYEVVLTTPSQLKQYYSSHTKDHKKLDSFGAGQYLVALLGECLGFQNGESWTRMRKSFNLFFTHTLAAKTLPAMIAFIDGWISEHDSQDEFSVDAFDFVATVPFTCIAKYLYGSEQCSGQVLTELKNLVPLHSELMTHAFTTFWGRFKIYQYFPFQRMKDLKFFQDSFKSLSLAMVESARDAENPTVASELYKLVESRDLTLDNWIQSLDEILFANIDVTATIMSWSLVEMGRNKHEQARLRLEVLENLQSVDEYCKRTDTVLHRVLLEILRLHPLLWYGFPEQSSSAMVIDGHKIEANTPIVVDQYQLNYKSPLWNPADKSTDYGATFDSDRFLGLNNRDILMSSVTFGSGPRRCLGKNFAEVLIKTEIAKVLSTFEVALEGELKVAADTFVVRPDAQIKLTRLI |
| yAMV642 | MLTILSLPVFWVLVVSLCLVSSKSKLRAFQKQPPFKKIPRSHRNVEGKKITQGPEVSEKNKNQYGSIYSHRDGFRYEVVLTTPSQLKQYYSSHTKDHKKLDSFGAGQYLVALLGECLGFQNGESWTRMRKSFNLFFTHTLAAKTLPAMIAFIDGWISEHDSQNEFSVDAFDFVATVPFTCIAKYLYGNEQCSGRVLTELKNLVPLHSELMTHAFTTFWGRFKLYQYFPFQRMKDLKFFQDSFKSLSLAMVESARDAENPTVASELYKLVESRDLTLDNWIQSLDEILFANIDVTATIMSWSLVEMGRNKHEQARLRLEVLENSQSVDEYCKRTDTVLHRVLLEILRLHPLLWYGFPEQSSSAMVIDGHKIEANTPIVVDQYQLNYKSPLWNPADKSTDYGATFDSDRFLGLNNRDILMSSVTFGSGPRRCLGKNFAEVLIKTEIAKVLSTFEVALEGELKVAADTFVVRPDAQIKLTRLI |
| *M. pulcherrima* APC1.2 | MITILSFPVFWVLVVSLCLVSSKSKLRAFQKQPPFKKIPRSHRNVEGKKITQGPEISEKNKNQYGSIYSHRDGFRYEVVLTTPSQLKQYYSSHTKDHKKLDSFGAGQYLVALLGECLGFQNGESWTRMRKSFNLFFTHTLAAKTLPAMIAFIDGWISEHDSQNEFSVDAFDFVATVPFTCIAKYLYGNEQCSGRILTELKNLVPLHSELMTHAFTTFWGRFKIYQYFPFQRMKDLKFFQDSFKSLSLAMVESARDAENPTVASELYKLVESRDLTLDNWIQSLDEILFANIDVTATIMSWSLVEMGRNKHEQARLRLEVLENLQSVDEYCKRTDTVLHRVLLEILRLHPLLWYGFPEQSSSAMVIDGHKIEANTPIVVDQYQLNYKSPLWNPADKRTDYGATFDSDRFLGLNNRDILMSSVTFGSGPRRCLGKNFAEVLIKTEIAKVLFTFEVALEGELKVAADTFVVRPDAQIKLTRLI |
| *M. pulcherrima* AP47 (*a*) | MLTILTFPVFWVLVVSLCLVSSKSKLRAFQKQPPFKKIPRSHRNVEGKKITQGPEISEKNKNQYGSIYSHRDGFRYEVVLTTPSQLKQYYSSHTKDHKKLDSFGAGQYLVALLGSVWAFKMVSCGLGCGNCLICFSHIRWSRKRCPR |
| *M. pulcherrima* AP47 (*b*) | MRKLFNLFFTHTLEPKTLPAMIAFIDGWISEHDSQNEFSVDAFDFVATVPFTCIAKYLYGNEQCSGRVLTELKNLVPLHSELMTHAFTTFWGRFKLYQYFPFQRMKDLKFFQDSFKSLSLAMVESARDAENPTVASELYKLVESRDLTLDNWIQSLDEILFANIDVTATIMSWSLVEMGRNKQEQGRLRLEVLENLQSVDEYCKRTDTVLHRVLLEILRLHPLLWYGFPEQSSSAMVIDGHKIEANTPIVVDQYQLNYKSPLWNPADKSTDYGATFDSDRFLGLNNRDILMSSVTFGSGPRRCLGKNFAEVLIKTEVAKVLSTFEVALEGELKVAADTFVVRPDAQIKLTRLI |
| *M. pulcherrima* KIOM G15050 | MITILSLPVFWVLVVSLCLVSSKSKLRAFQKQPPFKKIPRSHRNVEGKKITQGPEISEKNKNQYGSMYSHRDGFRYEVVLTTPSQLKQYYSSHTKDHKKLDSFGAGQYLVALLGECLGFQNGESWTRMRKSFNLFFTHTLAAKTLPAMIAFIDGWISEHDSQNEFSVDAFDFVATVPFTCIAKYLYGNEQCSGQVLTELKNLVPLHSELMTHAFTTFWGRFKIYQYFPFQRMKDLKFFQDSFKSLSLAMVESARDAENLTVASELYKLVESRDLTLDNWIQSLDEILFANIDVTATIMSWSLVEMGRNKHEQARLRLEVLENLQSVDEYCKRTDTVLHRVLLEILRLHPLLWYGFPEQSSSAMVIDGHKIEANTPIVVDQYQLNYKSPLWNPADKSTDYGATFDSDRFLGLNNRDILMSSVTFGSGPRRCLGKNFAEVLIKTEIAKVLSTFEVALEGELKVAADTFVVRPDAQIKLTRLI |
| *M. rubicola* CBS 15344 | MITILSFPVFWVLVVSLCLVSSKSQLRAFQKQPPFKKIPRSHRNVEGKKITQGPEISEKNKNQYGSIYSHRDGFRYEVVLTTPSQLKQYYSSHTKDHKKLDSFGAGQYLVALLGECLGFQNGESWTRMRKSFNLFFTHTLAAKTLPAMIAFIDGWISEHDSQDEFSVDAFDFVATVPFTCIAKYLYGNEQCSGRVLTELKNLVPLHSELMTHAFTTFWGRFKIYQYFPFQRMKDLKFFQDSFKSLSLAMVESARDAENPTVASELYKLVESRDLTLDNWIQSLDEILFANIDVTATIMSWSLVEMGRNKHEQARLRLEVLENLQSVDEYCKRSDTVLHRVLLEILRLHPLLWYGFPEQSSSAMVIDGHKIEANTPIVVDQYQLNYKSPLWNPADKSTDYGATFDSDRFLGLNNRDILMSSVTFGSGPRRCLGKNFAEVLIKTEIAKVLSTFEVALEGELKVAADTFVVRPDAQIKLTRLI |
| *M. chrysoperlae* NRRL Y–27615 | MITVLSFPVFWVLVVSLCLVTSKSKLRAFQKQPPFKKIPRSHRNVEGKKITEGPEISEKNKKQYGSIYSHRDGFRYEVVLTTPSQLKQYYSSHTKDHKKLDSFGAGQYLVALLGECLGFQNGESWTRMRNSFNLFFTHTLAAKTLPAMIAFIDGWISEHDSQNEFSVDAFDFVATVPFTCIAKYLYGDEQCSGRVLTELKNLVPLHSELMTHAFTTFWGRFKIYQYFPFQRMKDLKLFQDSFKSLSLAMVESARDAENPTVASELYKLVESRDLTLDNWIQSLDEILFANIDVTATIMSWSLVEMGRNRHEQVRLRLEVLENLQSVDEYCKRTDTVLHRVLLEILRLHPLLWYGFPEQSSSDMVIDGHKIEANTPIVVDQYQLNYKSPLWNPADKSTDYGATFDSDRFLGLNNRDILMSSVTFGSGPRRCLGKNFAEVLIKTEIAKVLSTFEVALEGELKVAADTFVVRPDAQIKLKRLI |
| *C. auris* B11221 | MSNLHVDLILVLVVATIFFSRKIIDQPNFSKLLKGSRNIPYTNRNVARYKLSKGSEISVENKSKLGTLYMHRDGFRYEVVLTTPDQLKQYFSSHPRDHAKLDSFGAGQYLVALLGECLGFQNGDSWMNMRKLFNGYFSHASAIQTLPTMVEFIGGYVESTTKESSKVVDPFEYVSAIPFTCIANYLYGKSHCTPERLSGLKELIPLHTDLLTFAFKTFWGRFKIFQFFQSDEMKGLRHFQDRFTQLSLEMVRNAGNDPSVASELYSRVEQGELSFDSWIQTLDEILFANIDVTATVMSWALVEMARNPNAQHAVREEIHSSTESQEEYVKKTETHLHRVLLETLRLHPLLWFGFPEVVSKDIVIDGFQIPAHTPIVIDQFQVNYESEIWNPPNKPKGFGHEFHPDRFIGLGNRDVLMSSVTFGSGPRRCLGKNFAEIVVKTLLVNMVKHFELSLCDPVEYAENTFVVQPKTKVKLGRIGV |
| *K. lactis* NRRL Y–1140 | MLADILIPLIKKNWMAFVYFTPVLFVVLYLLKEWRAAYGFNNLGQTVAAPFGYERKTLPYNKENCARTKFLDGKSLSIKNRDQCGDLYLQRSGTYKEIVLTTPKQLMEYYKSNSKNHSKLDSFGAGAFLVALLGECLGFQNGSEWLSMRKVFDSFFTHKAAVENFPVMIDYISEWIKDLDTEQISDIDPLQLVSDLPFTCIAKYLYGSELCSKQFLQALKDLIPMHTELMHYSFLTVAGRFKIFQYFPSKKMKQVSQFQRQFIDLSLKQVELSRQSGQETVVEKLYRHVESGKFTFNNWIQTIDEILFANIEVTSTVMAWALVEMGSNIEEQNRLRCEILKVKEQSSKDDFNKETDPMQRYMKLTDTYLQYCVWETLRMHPLLWFSFPEISSETLFIDGIRISPNTPIVVDQYQINYNSPIWNPSDKPKDFGKKFAPSRFENITLRDALYSQVTFGAGSRKCLGRNFAELLIKSELAYILSKYKVTLTEKVEFSKDTFVVQPKTKIQLTAL |
| **Pul3** | |
| yAMV511 | MAISQLVAIFASYCDFKIHNFKVNSNNGPTFVCSFIILAVTALLVFVLENPAVPTKKTSMSFTKALQGFFSAPKWTLAGGIILLWGMFFASFLMSEVVYFMPVFLTESLGWETKFQGVAFMVASIIGICGSLVFPHLIEMPIRKKEKEFNSQAESQSKLSEKSDISHNSEESADRKQEFEAYKKNALYNNQIVLSLVSLLIALVGQAFMIGAAQVFRHRSLPHINTGCFFVGGMSLVMLAYNGMASTFPALFSVYIDPQVKLQLMPAIGAVAALGKLIAPIVLSNLYQTKLGLSIAVGLGMILTALTLMPVFLLKDKKH |
| *M. pulcherrima* APC1.2 | MAISQLVAIFASYCDFKIHNFKVNSNNGPTFVCSFIILAVTALLVFVLENPAVPTKKTSLSFTKALQGFFSAPKWTLAGGIILLWGMFFASFLMSEVVYFMPVFLTESLGWETKFQGVAFMVASIIGICGSLVFPHLIEMPIRKKEKELNSQAESQSKLSEKSDISHNSEESADRKQEFEAYKKNALYNNQIVLSLVSLLIALVGQAFMIGAAQVFRHRSLPHINTGCFFVGGMSLVMLAYNGMASTFPALFSVYIDPQVKLQLMPAIGAVAALGKLIAPIVLSNLYQTKLGLSIAVGLGMILTALTLMPVFLLKDKKH |
| *M. pulcherrima* AP47 | MAISQLVAIFASYCDFKIHNFKVNSNNGPTFVCSFIILAVTALLVFVLENPAVPTKKTSMSFTKALQGFFSAPKWTLAGGIILLWGMFFASFLMSEVVYFMPVFLTESLGWETKFQGVAFMVASIIGICGSLVFPHLIEMPIRKKEKEFNSQAESQSKLSEKSDISHNSEESADRKQEFEAYKKKALYNNQIVLSLVSLLIALMGQAFMIGAAQVFRHRSLPHINTGCFFVGGMSLVMLAYNGMASTFPALFSVYIDPQVKLQLMPAIGAVAALGKLIAPIVLSNLYQTKLGLSIAVGLGMILTALTLMPVFLLKDKKH |
| *M. pulcherrima* KIOM G15050 | MAISQLVAIFASYCDFKIHNFKVNSNNGPTFVCSFIILAVTALLVFVLENPAVPTRKTRMSFTKALQGFFSAPKWTLAGGMILLWGMFFASFLMSEVVYFMPVFLTESLGWETKFQGVAFMIASIIGICGSLVFPHLIEMPIRKKEKEFNSQAESQSKLSEKSDISHNSEESADRKQEFEAYKKNALYNNQIVLSLVSLLIALVGQAFMIGAAQVFRHRSLPHINTGCFFVGGMSLVMLAYNGMASTFPALFSVYIDPQVKLQLMPAIGAVAALGKLIAPIVLSNLYQTKLGLSIAVGLGMILTALTLMPVFLLKDKKH |
| *M. rubicola* CBS 15344 | MAISQLVAIFASYCDFKIHNFKVNSNNGPTFVCSFIILAVTALLVFVLENPAVPTKKTSMSFTKALQGFFSAPKWTLGGGIILLWGMFFASFLMSEVVYFMPVFLTESLGWETKFQGVAFMVASIIGICGSLVFPHLIEMPIRKKEKEFNSQAESQSRLSEKSDISHNSEESADRKQEFEAYKKNALYNNQIVLSLVSLLIALVGQAFMIGAAQVFRHRSLPHINTGCFFVGGMSLVMLAYNGMASTFPALFSVYIDPQVKLQLMPAIGAVAALGKLIAPIVLSNLYQTKLGLSIAVGLGMILTALTLMPVFLLKDKKH |
| *M. chrysoperlae* NRRL Y–27615 | MAISQLVAIFASYCDFKIHNFKVNSNNGPTFVCSFIILAVTALLVFVLENPAVPTKKTSMSFTKALQGFFTAPKWTLAGGIILLWGMFFASFLMSEVVYFMPVFLTESLGWETKFQGVAFMVASIIGICGSLVFPHLIEMPIRKKEKELNAQAESQSKLSEKSDTSQNSEDSADRKQEFEAYKKNALYNNQVVLSLVSLLIALVGQAFMIGASQVFRHRSLPHINTGCFFVGGMSLVMLAYNGMASTFPALFSVYIDPQVKLQLMPAIGAVAALGKLIAPIVLSNLYQTKLGLSIAVGLGMILTALTLMPVFLLKIKKH |
| *C. auris* B11221 | MALSQLVAIFGSYCDFLIGDFRVSSNNAPTFITSFIMVLVAMGLFFTMKNPPVPKRKNTISFKRALLDFFTARRAQLLGSVIIMWGMFLASFLMSEVVYFMPVFLTQSLGWETKFQGVAFMVASLIGIIGSLLCPKLLSKLTENKEPEIHFESEKASTDESLEEMRKKQTEEFKKRQVFRNQVVLTLVSLAFALVGQAFMIGANEIFNDRRLPHINVGCFFVAGMSIIMLGYNGMASTFPSMFSEYVKPEVKVQLMPIIGAMTALGKLVAPIVLANLYTTRLGLSIAVALGIILTGITVPPTYCLIIS |
| *K. lactis* NRRL Y–1140 | MKLTDSQKHLYSQYLAVTLIAVQFSFDTCVYLSSVVQYVKECGSDDPENYLFILQAVSAAVQVFFSFIIGDIASYVGSIKWVIIFLYFLSFVGNFLYSCAGAVSLNTLLGGRIICGAASSSGAVVYSYITAISKDRTTIFKLFSIYRTSAGICMALAQLVAILFALCDFTVRGYRITSYNAPTFASSFIILLICVLLMFVLENPPVKSARNPKNYLDAWKKFFSAGSNRLIASLILLWNMFLSTFFMCEVLYFMPIFLTLNVGWKTEYEGVAFMVSAVLGVAGSFFAPDLVKLFAKLNTPSTQDETDTSDNDKIEKEESEQKSDINTLHRNQVSLTIFALFVALIGQAFMIGASEALSNDKLPKTNSGIFFTAGLSITMLGYNFMGSSVPALFSMYIDPQVKVQLMPFIGAIAGVGKLVAPIVLAALYKTPLGLPIGVGFGMILVGISIPSLVYLRRNKM |
| *S. cerevisiae* 288C | MSIAQDRGIVFKLLSIYRAAAGIFMALAQLIVIFFGYCDFKIKGYRIASYNAPTFASSFIILAVCLLLVVVLENPEVKVTNSENSLFSALKQFFRVERKKLISCLILLWSMFLSSFIMSEVVYFMPLFLTLHVNWDTKFQGIAFMVASILGVTGSYFAPKLINVGCSCGRAKDGGLEESDTTGSETVEVKKKDSLYSGQVFLSIFALFVSLLGQAFMIGASEALKHKSMPPTNSGIFFSAGMSITLLGYNFLASSIPALFSMYIDPKLKVQLMPSIGAISGIGKLVAPIVLAALYGTRLGLSIAVGFGMILVAVSIPPLIWLRKKRC |
| **Pul4** | |
| *M. pulcherrima* APC1.2 | MVKVAKTCTRCKSKKLKCDGSKPSCSRCLSLGITSCEYPPDKRVDKRQKLNGKDVFVFKNCDTTGDYKDIPETTLISPISQIEAGLDGSLSTFNASEFPTFFPSPDIGQQWPDFVPDLFQGDLNFSMDEFFCFLDDSVPLGSYDPLEQIEISPHKLLIDAVFSNKLHPPPGVSHEMILNIDMLQVRAEDEEFLHATILAMGALTLAKRDLVGQRQDTMVEVPEKIGLKLPAMASEAYGHYSRARELIPSMLKAPSRHGFRGLAVMANFMSILLTPETQMYISYHALQIAISIGLTQMSRVEINTEEYGLVIAFWGLWCSSCMLATFHGHAPPLMRSKISTTAHVQMKNRYVEVFFELRIKFAELSGIVAVVNKSEEINYQLLRKELSRLCEEISLFEESFPQEEIIGRHELFTLELKCWKAYVSMLLNLPALLEKRNTRAVVDAKNIVKDFWAYYYFYKRNFLPNLDWNFSYPLRNATLCVWFACMVLTRYINSSPALSFEFAEYKIGMELLIDLTGVIPINKYLIKELENVKNNELDSDKWLFKQG |
| *M. pulcherrima* AP47 | MVKVAKTCTRCKSKKLKCDGSKPSCSRCLSLGITSCEYPADKRVDKRQKLNGKDVFVFKNCDTTGDYKDIPETTLISPISQIEAGLDGSLSTFNASEFPTFFPSPDIGQQWPDFVPDLFQGDLNFSMDEFFCFLDDSVPLGSYDPLEQIEISPHKLLIDAVFSNKLHPPPGVSHEMILNIDMLQVRAEDEEFLHATILAMGALTLAKRDLVGQRQDTMVEVPEKVGLKLPAMASEAYGHYSRARELIPSMLKAPSRNGFRGLAVMANFMSILLTPETQMYISYHALQIAISIGLTQMSRVELDTEEYGLVIAFWGLWCSSCMLATFHGHAPPLMRSKISTTAHVQMKNRYVEVFFELRIKFAELSGIVAVVNKSEEINYQLLRKELSRLCEEISLFEESFPQEEIIGRHELFTLELKCWKAYVSMLLNLPALLEKRNSRAVVDAKNIVKDFWAYYYFYKRNFLPNLDWNFSYPLRNATLCVWFACMVLTRYINSSPALSFEFAEYKIGMELLTDLTGVIPINKYLIKELENVKNNELDSDKWLFKQG |
| *M. pulcherrima* KIOM G15050 (*a*) | MVKVAKTCTRCKLKKTQM |
| *M. pulcherrima* KIOM G15050 (*b*) | MASEAYGHYSRARELIPSMLKAPSRHGFRGLAVMANFMSILLTPETQMYISYHALQIAISIGLTQMSRVEIDTEEYGLVIAFWGLWCSSCMLATFHGHAPPLMRSKISTTAHVQMKNRYVEVFFELRIKFAELSGIVAVVNKSEEINYQLLRKELSRLCEEISLFEESFPQEEIIGRHELFTLELKCWKAYVSMLLNLPALLEKRNSRAVVDAKNIVKDFWAYYYFYKRNFLPNLDWNFSYPLRNATLCVWFACMVLTRYINSSPALSFEFAEYKIGMELLIDLTGVIPINKYLIKELENVKNNELDSDKWLFKQG |
| *M. rubicola* CBS 15344 | MVKVAKTCTRCKSKKLKCDGSKPSCSRCLSLGITSCEYPPDKRVDKRQKLNGKDVFVFKNSDTTGDHKDVPETTLISPISQIEAGLDASLSTFSATEFPTFFPSPDIGQQWPDFVPDLFQGDLNFSMDEFFCFLDDSVPLGSYDPLEQVEIPPPKLLLDAVFSNKLHPPPGVSYEMILNIDMLQVRAEDEEFLHATILAMGALTLAKRDLVGQRQDTMVEVPEKVGLKLPAMASEAYGHYSTARELIPSMLKAPSRHGFRGLAVMANFMSILLTPETQMYISYHALQIAISIGLTQVSRVEIDTEEYGLIIAFWGLWCSSCMLATFHGHAPPLMRSKITTTAHVQMKNRYVEIFFELRIKFAELSGIVAVVNKSEEINYQLLRKELSRLCEEISLFEESFPQEYIIGRHELFTLELKCWKAYVSMLLNLPALLEKRNSRAVVDAKNIVKDFWAYYYFYKRNFLPNLDWNFSYPLRNATLCVWFACMVLTTYINSSPALSFEFAEYKIGMELLTDLTGVIPINKYLIKELENVKNNQLEVDKWLFKQE |
| *M. chrysoperlae* NRRL Y–27615 | MVKVAKTCTRCKSKKLKCDGSKPSCSRCLSLGITSCEYPPDKRVDKRQKLNGKDVFVFKNSDTTGDHKDVPETTLISPISQIEAGLDASLSTFSATEFPTFFPSPDIGQQWPDFVPDLFQGDLNFSMDEFFCFLDDSVPLGSYDPLEQVEIPPPKLLLDAVFSNKLHPPPGVSYEMILNIDMLQVRAEDEEFLHATILAMGALTLAKRDLVGQRQDTMVEVPEKVGLKLPAMASEAYGHYSTARELIPSMLKAPSRHGFRGLAVMANFMSILLTPETQMYISYHALQIAISIGLTQVSRVEIDTEEYGLIIAFWGLWCSSCMLATFHGHAPPLMRSKITTTAHVQMKNRYVEIFFELRIKFAELSGIVAVVNKSEEINYQLLRKELSRLCEEISLFEESFPQEYIIGRHELFTLELKCWKAYVSMLLNLPALLEKRNSRAVVDAKNIVKDFWAYYYFYKRNFLPNLDWNFSYPLRNATLCVWFACMVLTTYINSSPALSFEFAEYKIGMELLTDLTGVIPINKYLIKELENVKNNQLEVDKWLFKQE |
| *C. auris* B11221 | MARACVTCKHKKLKCDGERPACGRCCKLGLPCEIPRDKRSDKRKVENGTSSFVFKNTGRKGRKHGWDKPVAIDDNELSSAPPLEASLGGEFVRGSLLEPTTLAWPELDEVKLPYDLDIEEFLHSLDYPLGLETTEPVGKVTSVFTGFSPVGEDQPTPYHVLIDTVFSDASHTPVSITKAMITGIADKVDRSEDESFLLSIVLAIGALTLAKQKYAEQRQSILKLGTDASDPSYHIKTPLVANEAVNHYQEARKLNSHILENPSAHGFRGLVLISNFLSGLLTLEAQMFISFHALQVAVAIKMHTNKETGSSEEESGLKIAFWELWCSACLFASFHGRLPPIRREEITTSLDFDLKNEYNKRFFHLRVEIAELHCQVAALKVNGLASSVSERDFHTKVSTISQRIVELEALFPQTESNFYRQELLTLELKCWMSQAIMLEGQQRLTRKLSIKSVVEARKLIRELWSYYNPEKFARGTMLSHLDWNFTYPLRTATIGAFTAAKTLTLFINSVDYLTFDFFDYCLSRKVLEFLTGVMSINKHLLQDLDNYALR |
| *K. lactis* NRRL Y–1140 | MACLECKKRKQKCDGQKPCRRCTKLNVKCIYGTDRRKDKRKIKDGSNMFIFKNQTLCNDKINGIVPHPLSHDTITTKETWEPSYPLFSDDINPADIISMENTDGSIPLQFDLDFTSLESCDVNDFLRLIGDTFPANDADTLDMNQMGGFNTPSISHNTQDDKSQIAIQRNRLIDVIFGDDSHTPPGILREHIFELSERHEDLEALDFDDNGKFLLSTVLCLGALTLRKRELLNRDSNQPSTNGIPEVAAGAYKYYTIATDLIPAVHAAPNIDGFCGLVLMANFMTIMIPLEGQLYLSNNALEVAVALNFHKRESYDEMIVSNPAQLGVFLLFWNLWCSSCMLATLLGKQPFLTLDNISLPPPHQMQHTVSSSPLSINFMRLRIQLATLQTKIFQRLYVYGSLNKVLFQEIETELSLLSTQISNMKCYPIYDEGLFYRSKVLMLELSCLKAHNAFLLYRPNLIQKKSLHAVDAAKHIILEIWSHYTKQFPKNEKDLVDHLDWNFSYPLRTASLTLSISCVILQKYQQSLNFLEEYGIFEYNLALGVLNDLIQVVPIEKRLINLLTVSRTTVEDANESNREDSLRFWTNMLMC |
| *S. cerevisiae* 288C | MDRSKDARKRSISLACTVCRKRKLKCDGNKPCGRCIRLNTPKECIYNIDKRKDKRKIKNGSKVFLFKNNTIDNGNNSILENKGLNEDLSSHIYEKEAPKFDSDIDISRFGTNDAVIFNNDGWDTSLPIDFDFDEFNTETTDFDDFLKLLGDNSPSKEQKSLSYSPTATGLSGVVKETESEDNAPTRSRLIDVLFENKLHSVPGISKWHLYELESQYPNLECTEGNSDEKFLLSTVLCLGSLTIRKRELLNHSNIDNRPLLPENSISKLTTDAFKYYNAAKTLVPDLLSHPTIDGFCGLVLMANFMTMMISLEHQLYLSINALQLAVALNLNNNTKCKELLESNSDGIGVILLFWNIWCSSCMLATIHGKNPFITLEQITTPLPCEISPRNKTNKLLIDFMQIRIKLATLQSKIFQRLYTSSTANEVPFVNLEREFEEVSLQITRLKGFPIFEEHLFYRSRVLMLELSCLRAQASFLLYRPYLITGESLQAVTMAKSIIHEIWSQYTKQFPDNEKERHERLDWNFCYPLRTASLTLCISCIILLRYKQVVQFLKGTELFEYILALEILQDLVQVLPIEQNLIDIIKYPISPVQLSGDSFVEFWGRILY |

**Supplementary Table 7.** Percentage identity matrix for Pul1 primary protein sequences from genome–available *K. lactis, C. auris* and *Metschnikowia* species reported to produce iron chelators and *Metschnikowia* isolates from this study. ‘Kl’ refers to *Kluyveromyces lactis* NRRLY–1140, ‘Ca’ refers to *Candidozyma auris* B11221, ‘Mc’ refers to *Metschnikowia chrysoperlae* NRRLY–27615*,* ‘Mr’ refers to *Metschnikowia rubicola* CBS15344, ‘Mp1’ refers to *Metschnikowia pulcherrima* APC1.2 and ‘Mp2’ refers to *Metschnikowia pulcherrima* AP47.

|  | **Kl** | **Ca** | **Mc** | **Mr** | **Mp2** | **Mp1** | **yAMV511** | **yAMV312** | **yAMV636** | **yAMV240** | **yAMV460** | **yAMV642** |
| --- | --- | --- | --- | --- | --- | --- | --- | --- | --- | --- | --- | --- |
| **Kl** |  | 30.77 | 30.91 | 30.23 | 30.68 | 30.45 | 30.45 | 30.45 | 30.68 | 30.45 | 30.68 | 30.45 |
| **Ca** | 30.77 |  | 41.9 | 41.67 | 41.67 | 41.2 | 42.13 | 41.67 | 41.67 | 41.44 | 41.44 | 41.67 |
| **Mc** | 30.91 | 41.9 |  | 88.86 | 88.86 | 89.74 | 89.52 | 89.3 | 89.3 | 89.52 | 89.74 | 89.96 |
| **Mr** | 30.23 | 41.67 | 88.86 |  | 97.38 | 97.38 | 98.03 | 97.82 | 98.03 | 98.03 | 98.03 | 98.25 |
| **Mp2** | 30.68 | 41.67 | 88.86 | 97.38 |  | 97.38 | 97.6 | 98.25 | 97.6 | 97.82 | 98.47 | 98.69 |
| **Mp1** | 30.45 | 41.2 | 89.74 | 97.38 | 97.38 |  | 97.38 | 97.82 | 98.47 | 98.47 | 98.47 | 98.69 |
| **yAMV511** | 30.45 | 42.13 | 89.52 | 98.03 | 97.6 | 97.38 |  | 98.03 | 98.47 | 97.6 | 98.47 | 98.69 |
| **yAMV312** | 30.45 | 41.67 | 89.3 | 97.82 | 98.25 | 97.82 | 98.03 |  | 97.6 | 97.6 | 98.47 | 98.69 |
| **yAMV636** | 30.68 | 41.67 | 89.3 | 98.03 | 97.6 | 98.47 | 98.47 | 97.6 |  | 98.69 | 98.69 | 98.91 |
| **yAMV240** | 30.45 | 41.44 | 89.52 | 98.03 | 97.82 | 98.47 | 97.6 | 97.6 | 98.69 |  | 98.69 | 98.91 |
| **yAMV460** | 30.68 | 41.44 | 89.74 | 98.03 | 98.47 | 98.47 | 98.47 | 98.47 | 98.69 | 98.69 |  | 99.78 |
| **yAMV642** | 30.45 | 41.67 | 89.96 | 98.25 | 98.69 | 98.69 | 98.69 | 98.69 | 98.91 | 98.91 | 99.78 |  |

**Supplementary Table 8.** Percentage identity matrix for Pul2 primary protein sequences from genome–available *K. lactis, C. auris* and *Metschnikowia* species reported to produce iron chelators and *Metschnikowia* isolates from this study. ‘Kl’ refers to *Kluyveromyces lactis* NRRLY–1140, ‘Ca’ refers to *Candidozyma auris* B11221, ‘Mc’ refers to *Metschnikowia chrysoperlae* NRRLY–27615*,* ‘Mr’ refers to *Metschnikowia rubicola* CBS15344, ‘Mp1’ refers to *Metschnikowia pulcherrima* APC1.2 and ‘Mp3’ refers to *Metschnikowia pulcherrima* KIOM G15050.

|  | **Kl** | **Ca** | **Mc** | **yAMV**  **240** | **yAMV**  **511** | **yAMV**  **642** | **yAMV**  **460** | **Mp1** | **yAMV**  **636** | **Mp3** | **yAMV**  **312** | **Mr** |
| --- | --- | --- | --- | --- | --- | --- | --- | --- | --- | --- | --- | --- |
| **Kl** |  | 45.89 | 50 | 51.26 | 50.42 | 50.42 | 50.21 | 50.21 | 51.05 | 50.63 | 50.42 | 50.21 |
| **Ca** | 45.89 |  | 56.78 | 56.57 | 56.36 | 56.36 | 56.78 | 56.78 | 56.78 | 56.57 | 56.57 | 56.57 |
| **Mc** | 50 | 56.78 |  | 96.46 | 96.67 | 96.67 | 96.88 | 97.08 | 96.88 | 96.88 | 97.29 | 97.08 |
| **yAMV**  **240** | 51.26 | 56.57 | 96.46 |  | 98.12 | 97.92 | 97.71 | 97.92 | 98.33 | 97.71 | 98.12 | 97.92 |
| **yAMV**  **511** | 50.42 | 56.36 | 96.67 | 98.12 |  | 98.54 | 98.33 | 98.12 | 98.54 | 98.33 | 98.33 | 98.12 |
| **yAMV**  **642** | 50.42 | 56.36 | 96.67 | 97.92 | 98.54 |  | 98.54 | 98.33 | 98.75 | 98.54 | 98.54 | 98.33 |
| **yAMV**  **460** | 50.21 | 56.78 | 96.88 | 97.71 | 98.33 | 98.54 |  | 99.79 | 98.54 | 98.75 | 98.75 | 98.54 |
| **Mp1** | 50.21 | 56.78 | 97.08 | 97.92 | 98.12 | 98.33 | 99.79 |  | 98.33 | 98.54 | 98.96 | 98.75 |
| **yAMV**  **636** | 51.05 | 56.78 | 96.88 | 98.33 | 98.54 | 98.75 | 98.54 | 98.33 |  | 98.96 | 98.96 | 98.75 |
| **Mp3** | 50.63 | 56.57 | 96.88 | 97.71 | 98.33 | 98.54 | 98.75 | 98.54 | 98.96 |  | 99.17 | 98.54 |
| **yAMV**  **312** | 50.42 | 56.57 | 97.29 | 98.12 | 98.33 | 98.54 | 98.75 | 98.96 | 98.96 | 99.17 |  | 99.38 |
| **Mr** | 50.21 | 56.57 | 97.08 | 97.92 | 98.12 | 98.33 | 98.54 | 98.75 | 98.75 | 98.54 | 99.38 |  |


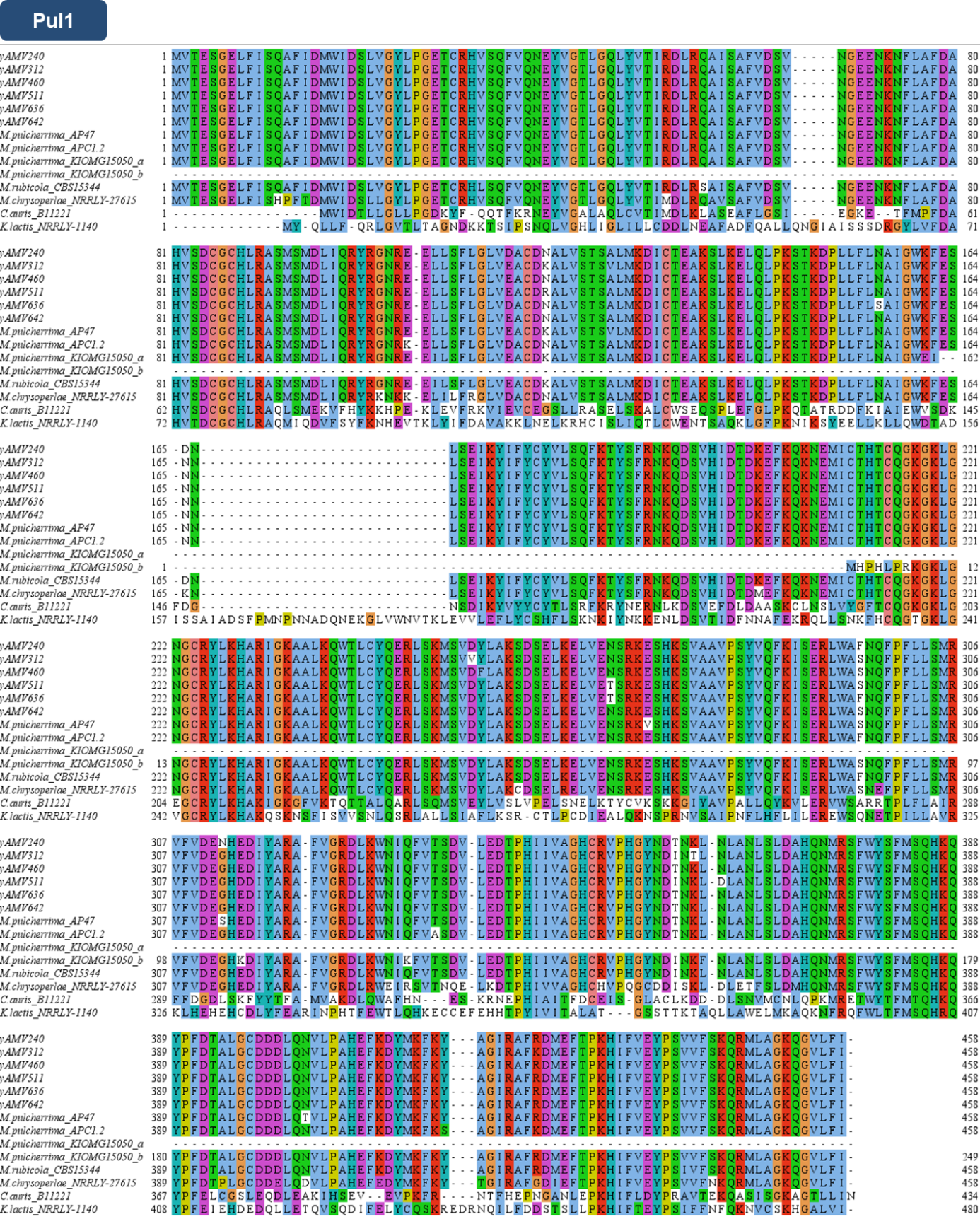


**Supplementary Figure 1**. Multiple sequence alignment of Pul1 from Metschnikowia species, C. auris and K. lactis from NCBI databank and yeast isolates from this study. Amino acids are highlighted using Clustal Colour Scheme. All Metschnikowia strains show high similarity but a unique Pul1 sequence. For M. pulcherrima KIOM G15050, two ORFs were identified that aligned to different parts of the Pul1 proteins of other organisms, shown as a and b.


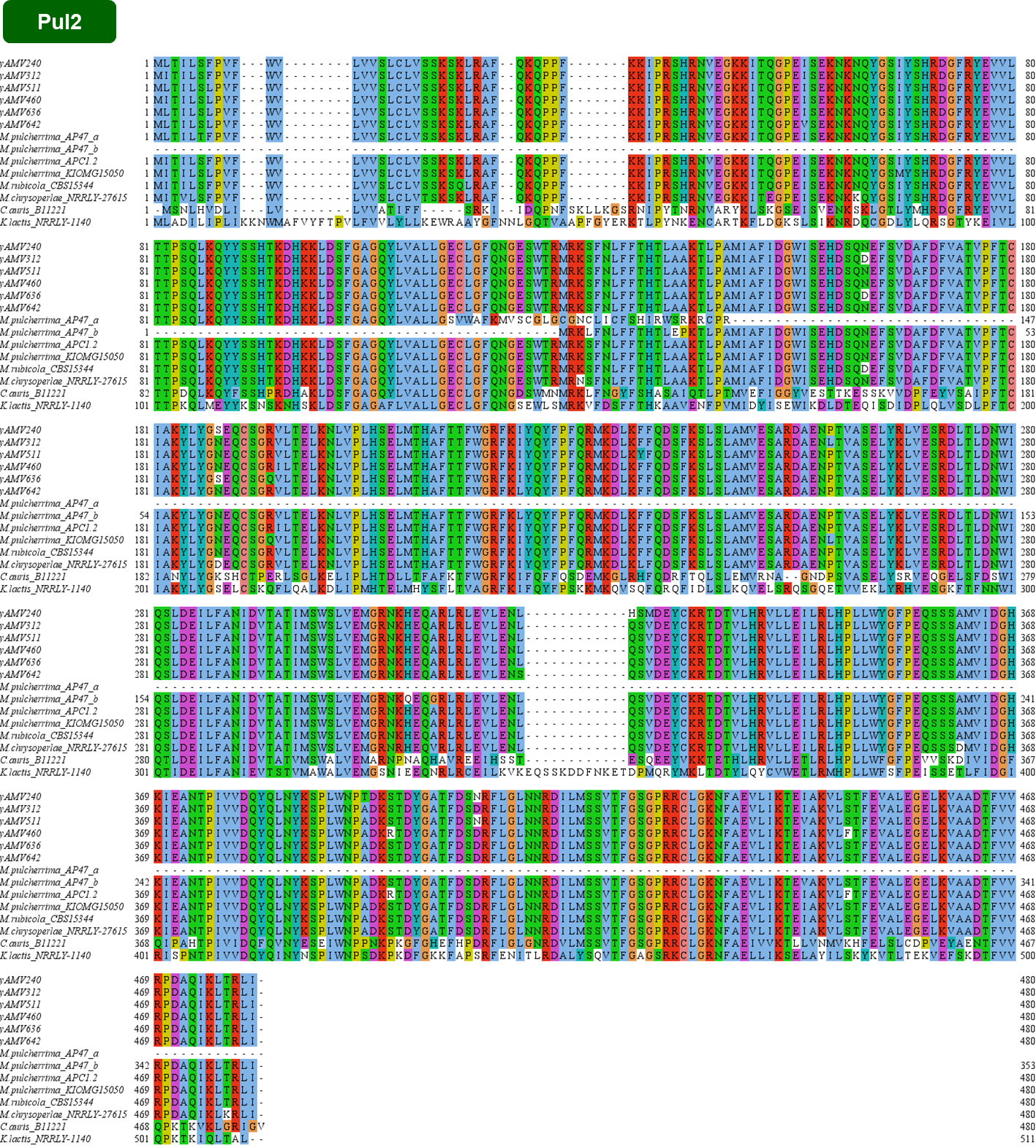


**Supplementary Figure 2.** Multiple sequence alignment of Pul2 from Metschnikowia species, K. lactis and C. auris from NCBI databank and yeast isolates from this study. Amino acids are highlighted using Clustal Colour Scheme. All Metschnikowia strains show high similarity but a unique Pul2 primary protein sequence. For M. pulcherrima AP47, two ORFs were identified that aligned to different parts of the Pul2 proteins of other organisms, shown as a and b.


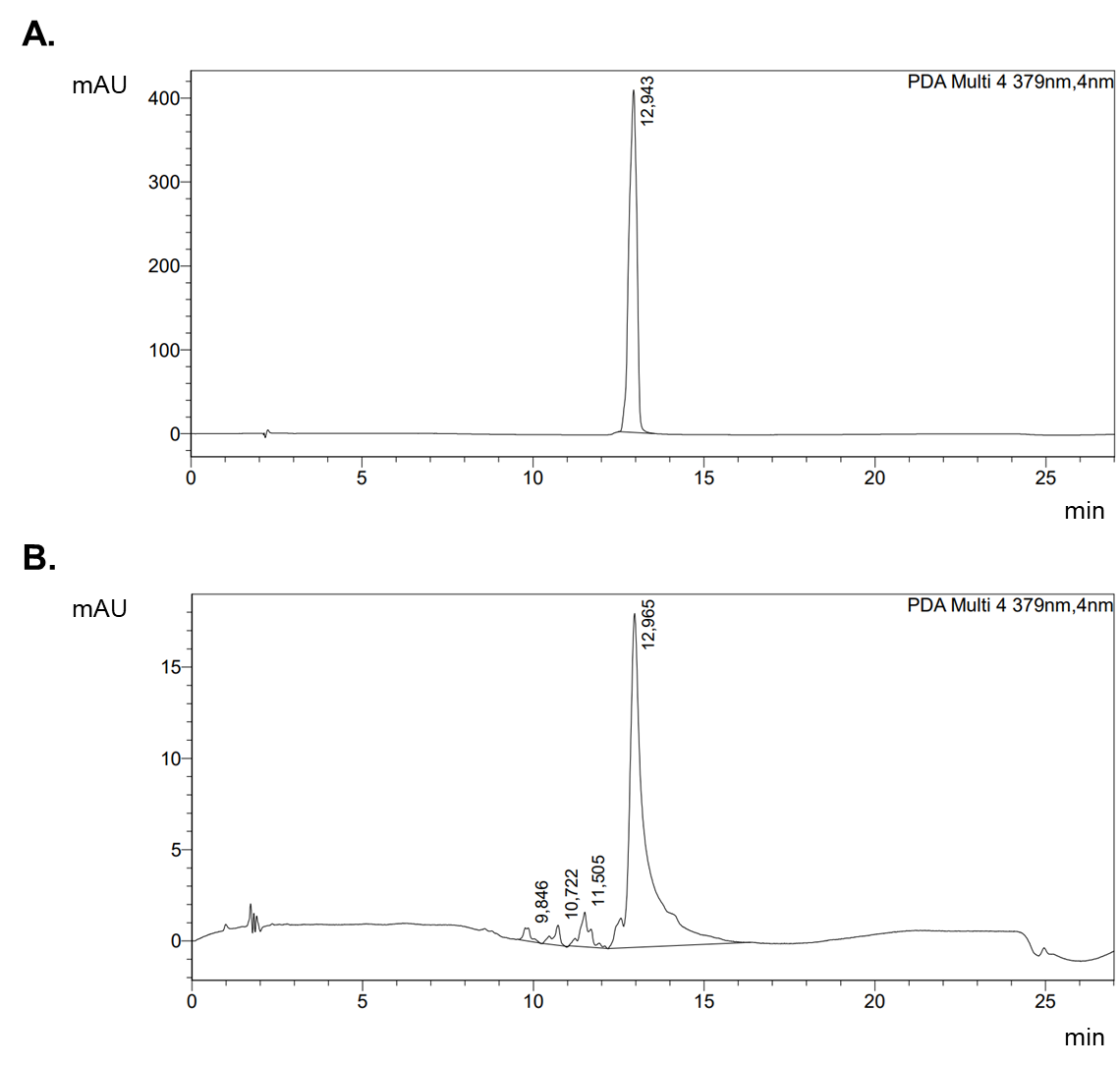


**Supplementary Figure 3**. HPLC chromatogram of (**A**) pulcherriminic acid standard dissolved in DMSO and (**B**) 50–fold concentrated spent media from yAMV511 isolate culture at 379 nm. Both samples show a pick at the same retention time.


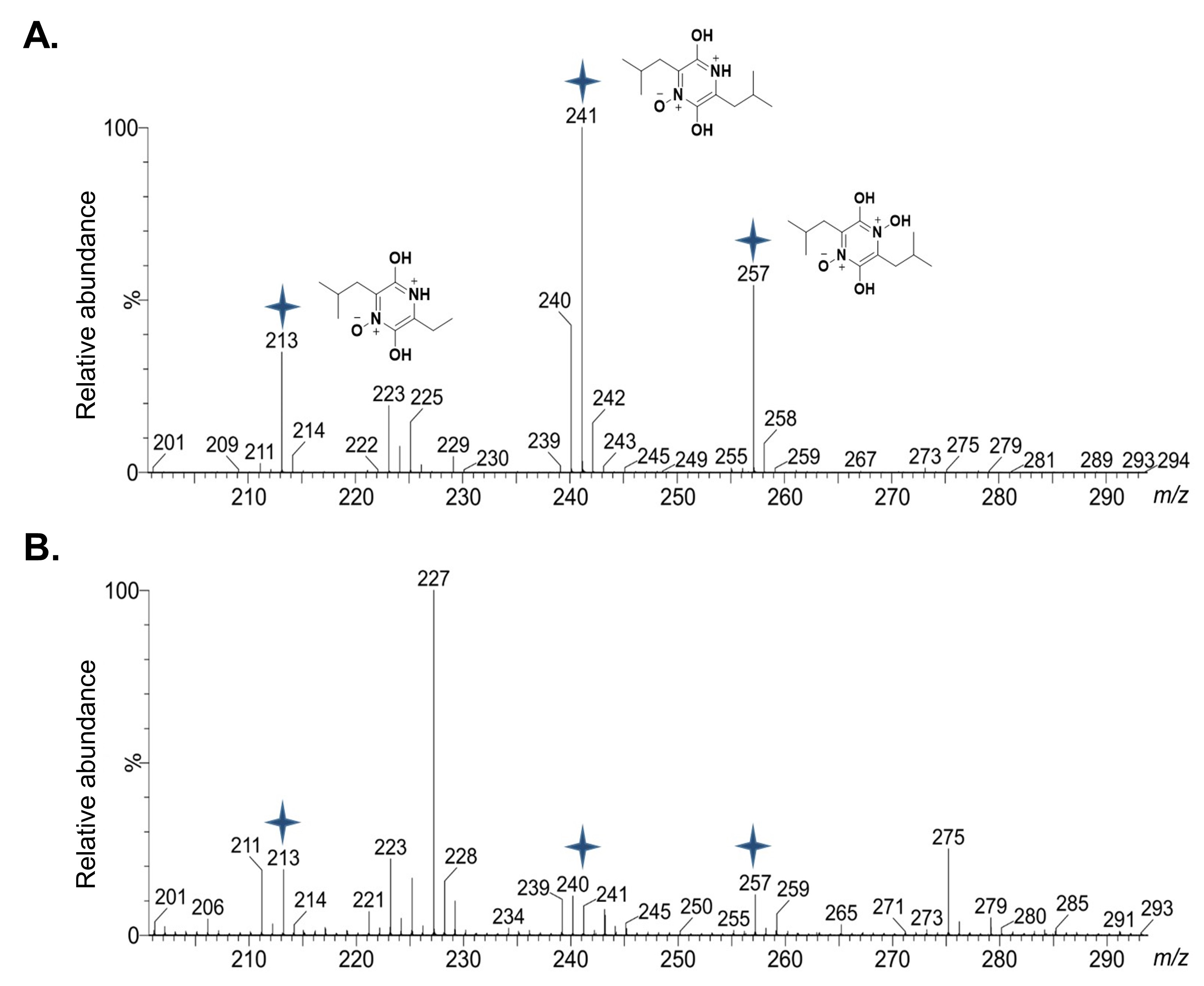


**Supplementary Figure 4.** Mass spectrometry profile of (**A**) pulcherriminic acid standard dissolved in DMSO and (**B**) 50–fold concentrated spent media from yAMV511 isolate culture. The peaks with highest intensity in the standard (highlighted and with the chemical structure) can be found in the spent media of yAMV511 as well, indicating pulcherriminic acid production.

**References**

Lee ME, DeLoache WC, Cervantes B *et al.* A Highly Characterized Yeast Toolkit for Modular, Multipart Assembly. *ACS Synth Biol* 2015;**4**:975–86.

Maciá Valero A, Tabatabaeifar F, Billerbeck S. Screening a 681-membered yeast collection for the secretion of proteins with antifungal activity. *N Biotechnol* 2025;**86**:55–72.

O´Donnell K. Fusarium and its near relatives. *Fungal Holomorph Mitotic, Meiotic Pleomorphic Speciat Fungal Syst* 1993:225–33.

White TJ, Bruns T, Lee S *et al.* Amplification and Direct Sequencing of Fungal Ribosomal Rna Genes for Phylogenetics. *PCR Protoc* 1990:315–22.
